# Preclinical Characterization and Phase I Trial Results of a Bispecific Antibody Targeting PD-L1 and 4-1BB (GEN1046) in Patients with Advanced Refractory Solid Tumors

**DOI:** 10.1158/2159-8290.CD-21-1345

**Published:** 2022-02-17

**Authors:** Alexander Muik, Elena Garralda, Isil Altintas, Friederike Gieseke, Ravit Geva, Eytan Ben-Ami, Corinne Maurice-Dror, Emiliano Calvo, Patricia M. LoRusso, Guzman Alonso, Maria E. Rodriguez-Ruiz, Kristina B. Schoedel, Jordan M. Blum, Bianca Sänger, Theodora W. Salcedo, Saskia M. Burm, Eliana Stanganello, Dennis Verzijl, Fulvia Vascotto, Angelica Sette, Juliane Quinkhardt, Theo S. Plantinga, Aras Toker, Edward N. van den Brink, Mark Fereshteh, Mustafa Diken, David Satijn, Sebastian Kreiter, Esther C.W. Breij, Gaurav Bajaj, Eleni Lagkadinou, Kate Sasser, Özlem Türeci, Ulf Forssmann, Tahamtan Ahmadi, Uğur Şahin, Maria Jure-Kunkel, Ignacio Melero

**Affiliations:** 1 BioNTech, Mainz, Germany.; 2 Medical Oncology Department, Vall d'Hebron University Hospital and Institute of Oncology, Barcelona, Spain.; 3 Genmab B.V., Utrecht, the Netherlands.; 4 Oncology Division, Tel Aviv Sourasky Medical Center, Tel Aviv, Israel.; 5 Department of Oncology, Chaim Sheba Medical Center, Ramat Gan, Israel.; 6 Institute of Oncology, Rambam Health Care Campus, Haifa, Israel.; 7 START Madrid-CIOCC, Clara Campal Comprehensive Cancer Center, Madrid, Spain.; 8 Yale Cancer Center, Yale University, New Haven, Connecticut.; 9 Radiation Oncology Department, Clínica Universidad de Navarra, Pamplona, Spain.; 10 Genmab U.S. Inc., Princeton, New Jersey.; 11 TRON gGmbH, Translational Oncology at the University Medical Center of the Johannes Gutenberg University, Mainz, Germany.; 12 Genmab A/S, Copenhagen, Denmark.; 13 Department of Immunology, Clínica Universidad de Navarra and CIBERONC, Pamplona, Spain.

## Abstract

Checkpoint inhibitors (CPI) have revolutionized the treatment paradigm for advanced solid tumors; however, there remains an opportunity to improve response rates and outcomes. In preclinical models, 4-1BB costimulation synergizes with CPIs targeting the programmed cell death protein 1 (PD-1)/programmed cell death ligand 1 (PD-L1) axis by activating cytotoxic T-cell–mediated antitumor immunity. DuoBody-PD-L1×4-1BB (GEN1046) is an investigational, first-in-class bispecific immunotherapy agent designed to act on both pathways by combining simultaneous and complementary PD-L1 blockade and conditional 4-1BB stimulation in one molecule. GEN1046 induced T-cell proliferation, cytokine production, and antigen-specific T-cell–mediated cytotoxicity superior to clinically approved PD-(L)1 antibodies in human T-cell cultures and exerted potent antitumor activity in transplantable mouse tumor models. In dose escalation of the ongoing first-in-human study in heavily pretreated patients with advanced refractory solid tumors (NCT03917381), GEN1046 demonstrated pharmacodynamic immune effects in peripheral blood consistent with its mechanism of action, manageable safety, and early clinical activity [disease control rate: 65.6% (40/61)], including patients resistant to prior PD-(L)1 immunotherapy.

**Significance:**

DuoBody-PD-L1×4-1BB (GEN1046) is a first-in-class bispecific immunotherapy with a manageable safety profile and encouraging preclinical and early clinical activity. With its ability to confer clinical benefit in tumors typically less sensitive to CPIs, GEN1046 may fill a clinical gap in CPI-relapsed or refractory disease or as a combination therapy with CPIs.

*See related commentary by Li et al., p. 1184 .*

*This article is highlighted in the In This Issue feature, p. 1171*

## Abstract (precis)

GEN1046, which combines simultaneous PD-1/PD-L1 blockade and conditional 4-1BB stimulation, shows encouraging preclinical results and in a first-in-human dose-escalation study, demonstrates manageable safety and clinical activity in patients with advanced solid tumors.

## INTRODUCTION

Immune checkpoint inhibitors (CPI) targeting the programmed cell death protein 1 (PD-1) receptor and its ligand, programmed cell death ligand 1 (PD-L1), have transformed cancer treatment to become the standard of care in various advanced solid malignancies (1, 2). However, depending on the tumor type, many patients do not respond or respond only transiently to anti–PD-1/PD-L1 [PD-(L)1] monotherapy (3–8). Novel therapeutic modalities are needed to stimulate effective and durable antitumor immunity for patients with PD-(L)1–refractory disease. One potential strategy is to develop regimens targeting complementary immunoregulatory pathways able to enhance efficacious immune responses against tumors (9).

The TNF receptor superfamily member 9 (CD137 or 4-1BB) is an inducible T-cell costimulatory receptor expressed on the surface of activated CD4^+^ and CD8^+^ T cells and activated natural killer (NK) cells (9–11). Costimulation of 4-1BB has the potential to complement and enhance antitumor activity of PD-1/PD-L1 blockade by improving functionality and survival of chronically stimulated CD8^+^ T cells in the tumor microenvironment (TME; refs. 12, 13) and expanding T-cell clonality (14) and the overall prevalence of T cells (15). Tumor antigen–specific T cells express PD-1 and 4-1BB, providing a further rationale for cotargeting these pathways to expand tumor-specific T-cell responses (16, 17).

Agonistic 4-1BB mAbs were validated as promising cancer immunotherapies in preclinical and early clinical studies (9, 18). However, despite promising initial efficacy signals, further clinical development was hindered by a limited therapeutic window (9, 19). Urelumab, a strongly agonistic 4-1BB mAb, demonstrated promising antitumor activity in phase I/II trials, but further development as a monotherapy was hampered by dose-limiting hepatotoxicity characterized by elevations in serum transaminase levels (20–22). By contrast, utomilumab, a weak 4-1BB agonistic mAb, demonstrated better hepatic safety but limited antitumor activity as monotherapy (23). Combination approaches and next-generation agents are being developed with the goal of broadening the therapeutic window of 4-1BB–targeting agents (18).

Bispecific antibody (bsAb) technologies provide opportunities to generate immunotherapy agents with superior or novel properties beyond those of the respective individual mAbs or their mixtures. DuoBody-PD-L1×4-1BB (GEN1046), a bsAb targeting PD-L1 and 4-1BB, is a next-generation checkpoint immunotherapy designed to enhance T-cell and NK-cell function through conditional 4-1BB stimulation while constitutively blocking the PD-1/PD-L1 inhibitory axis. Herein we describe the generation and preclinical characterization of GEN1046 and results from the ongoing first-in-human study evaluating GEN1046 in heavily pretreated patients with advanced solid tumors.

## RESULTS

### Generation of GEN1046 and Target-Binding Characteristics

GEN1046 is a full-length IgG1 PD-L1×4-1BB bsAb created via Fab-arm exchange of monoclonal fully human PD-L1 and humanized 4-1BB antibodies using the DuoBody technology platform (Supplementary Fig. S1A), which generates stable antibodies that retain native IgG structure and pharmacokinetics (24, 25). The parental PD-L1– and 4-1BB–specific antibodies for GEN1046 were selected based on their biological activity in the bispecific format *in vitro*. Binding of IgG to Fcγ receptors and complement C1q was abrogated to avoid 4-1BB clustering on the plasma membrane and activation through Fcγ receptor–mediated cross-linking of target-bound antibody (Supplementary Fig. S1B). The PD-L1– and 4-1BB–specific Fab arms of GEN1046 bound to its targets with affinities in the subnanomolar range (*K*_D_ PD-L1: 0.16 nmol/L, 4-1BB: 0.15 nmol/L) determined by biolayer interferometry, showed dose-dependent binding to primary human PD-L1^+^ or 4-1BB^+^ cells and blocked binding of 4-1BB to its natural ligand, TNF superfamily member 9 (4-1BBL; Supplementary Fig. S1C–S1E).

### GEN1046 Promotes Interactions between Dendritic Cells and T Cells and Enhances T-cell Activation

**Figure 1.**
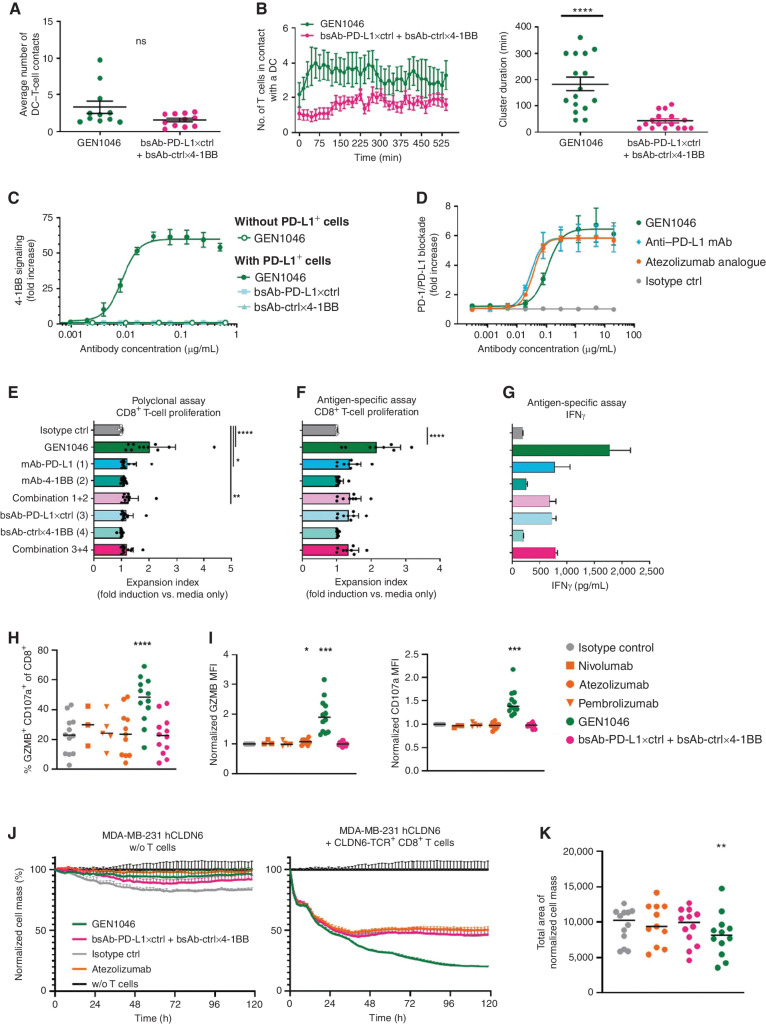
GEN1046 induces dose-dependent, conditional T-cell proliferation and cytokine production and enhances antigen-specific T-cell–mediated cytotoxicity *in vitro*. **A** and **B,** Activated T cells were cocultured with autologous iDCs in the presence of GEN1046 or control antibodies (0.125 µg/mL), and T-cell/iDC clusters were visualized over time by live-cell imaging. Quantification of the number of T cells in contact with a given DC on average (**A**), or over time (**B**; left), as well as the duration of these DC/T-cell clusters (**B**; right) is shown. ****, *P* < 0.0001; ns, not significant; Mann–Whitney *U* test. **C,** Induction of 4-1BB signaling by GEN1046 or control antibodies was assessed using a 4-1BB reporter assay. **D,** Blockade of the PD-1/PD-L1 interaction by GEN1046 or control antibodies was assessed using a PD-1/PD-L1 blockade bioassay. **E,** CFSE-labeled human PBMCs were stimulated with anti-CD3 (0.1 µg/mL) and incubated with GEN1046 or control antibodies (0.2 µg/mL) for 4 days. CFSE dilution in CD8^+^ T cells was analyzed by flow cytometry, and the expansion index was calculated. Data shown are the fold change in expansion index of treatment groups, relative to untreated cells of individual donors, as well as mean ± standard deviation (*n* = 11). ****, *P* < 0.0001; **, *P* < 0.01; *, *P* < 0.05, Friedman test with Dunn multiple comparisons test. **F,** CD8^+^ T cells were electroporated with RNA encoding a CLDN6-specific TCR and PD-1, labeled with CFSE, and cocultured with autologous DCs electroporated with CLDN6-encoding RNA in the presence of GEN1046 or control antibodies (0.2 µg/mL) for 4 days. Proliferation was measured by CFSE dilution as described in **E**. ****, *P* < 0.0001, Friedman test with Dunn multiple comparisons test. **G,** IFNγ concentrations in supernatant taken after 48 hours from cultures as described in **F**. Data shown are mean concentration ± standard deviation of triplicate wells from one representative donor (*n* = 3 donors). **H–K,** CD8^+^ T cells were electroporated with RNA encoding a CLDN6-specific TCR and were preactivated for 24 hours in coculture with CLDN6-expressing MDA-MB-231 cells (MDA-MB-231 hCLDN6) to induce 4-1BB expression. Subsequently, the preactivated CD8^+^ T cells were transferred to cocultures with previously seeded MDA-MB-231 hCLDN6 tumor cells (hCLDN6^+^ PD-L1^+^) in the presence of GEN1046 or control antibodies (0.2 µg/mL). **H**–**I,** After 48 hours, expression of cytotoxic mediator GZMB and degranulation marker CD107a by the CD8^+^ T cells was assessed; pooled data from four experiments (*n* = 3–12) are depicted. MFIs are expressed as relative values compared with the isotype control condition. **J,** Cytotoxicity of the CD8^+^ T cells toward MDA-MB-231 CLDN6 cells was monitored by electrical impedance measurement over 5 days using the xCELLigence real-time cell analysis. Representative data (mean ± standard deviation of triplicate wells) from cocultures derived from one individual donor are shown (*n* = 12). Data were normalized to the time point of coculture start and expressed relative to tumor-cell cultures without T cells (without T cells set to 100%). **K,** AUC (total area) analysis of cytotoxicity data (*n* = 11–12). ****, *P* < 0.0001; ***, *P* < 0.001; **, *P* < 0.01; *, *P* < 0.05, mixed-effect analysis with Dunnett multiple comparisons test. MFI, median fluorescence intensity; ns, not significant.

Analyses of cell–cell interactions by flow cytometry demonstrated that GEN1046 simultaneously binds to PD-L1– and 4-1BB–expressing cells over a broad range of concentrations, with maximal performance between 0.01 and 1 µg/mL, as compared with a combination of monovalent-control bsAbs (Supplementary Fig. S2A). Fluorescence microscopy studies showed that GEN1046 increased the number of T-cell contacts per dendritic cell (DC; Fig. 1A). Furthermore, live-cell imaging demonstrated that GEN1046 significantly prolonged the duration of DC/T-cell engagement (Fig. 1B). GEN1046 colocalized with integrin subunit α L (LFA1; Supplementary Fig. S2B and S2C), a marker of immunologic synapses between T cells and antigen-presenting cells (26). In a cell-based reporter assay for 4-1BB activation using Jurkat cells stably transfected with NFκB inducible luciferase and 4-1BB, GEN1046 activated 4-1BB signaling upon coculture with PD-L1–expressing cells, whereas 4-1BB signaling was not observed in the absence of PD-L1–expressing cells (Fig. 1C). Similarly, the 4-1BB–binding monovalent-control bsAb did not induce 4-1BB signaling, indicating that the 4-1BB agonist activity of GEN1046 was strictly conditional (i.e., dependent on cross-linking to PD-L1^+^ cells). By blocking the PD-1/PD-L1 interaction, GEN1046 reversed inhibition of T-cell receptor (TCR) signaling in a cell-based reporter assay for inhibition of the PD-1/PD-L1 pathway (Fig. 1D). Maximal blocking activity was comparable with bivalent monoclonal PD-L1–blocking antibodies, but it was reached at slightly higher concentrations of GEN1046 than the reference PD-(L)1–blocking antibodies. GEN1046 also blocked the PD-1/PD-L1 axis in the absence of 4-1BB binding, showing that the PD-L1–specific Fab arm of GEN1046 also functions as a classic immune CPI.

T-cell proliferation was analyzed in anti-CD3–stimulated peripheral blood mononuclear cells (PBMC; polyclonal assay) or cocultures of autologous PD-L1^+^ immature DCs (iDC) engineered to express claudin 6 (CLDN6) and CLDN6-TCR^+^ CD8^+^ T cells (antigen-specific assay). GEN1046 significantly enhanced the proliferation of activated CD8^+^ T cells in polyclonal (Fig. 1E) and antigen-specific settings (Fig. 1F). Similarly, GEN1046 conditionally enhanced the proliferation of anti-CD3–stimulated CD4^+^ T cells from the same donors, although to a lesser extent compared with CD8^+^ T cells (Supplementary Fig. S2D). Importantly, GEN1046 specifically induced proliferation in activated T cells, as no effect on the proliferation of CD8^+^ or CD4^+^ T cells was observed in the absence of CD3 or TCR stimulation (Supplementary Fig. S2E and S2F). CD8^+^ T-cell proliferation induced by GEN1046 was dose-dependent, with half-maximal effective concentration values in the picomolar range for the polyclonal (39 ± 10 pmol/L) and antigen-specific (15 ± 2 pmol/L; Supplementary Fig. S2G and S2H) assays. GEN1046 strongly and dose dependently enhanced production of proinflammatory cytokines, most prominently IFNγ, in antigen-specific *in vitro* assays (Fig. 1G; Supplementary Fig. S2I). In these primary T-cell assays, GEN1046-induced increases in T-cell proliferation and cytokine secretion superior to PD-L1 blockade alone and the combination of PD-L1–targeting and 4-1BB–targeting antibodies, indicating that full T-cell stimulation induced by GEN1046 was conditional (i.e., dependent) on simultaneous binding to PD-L1 and 4-1BB.

### GEN1046 Induces T-cell–Mediated Cytotoxicity of PD-L1^+^ Tumor Cells

We evaluated the capacity of GEN1046 to enhance T-cell–mediated cytotoxicity of PD-L1–expressing tumor cells by coculturing CLDN6-TCR^+^ CD8^+^ T cells (preactivated to induce 4-1BB expression) with CLDN6^+^ PD-L1^+^ MDA-MB-231 target cells in the presence of GEN1046 or clinical-grade PD-(L)1–blocking agents. GEN1046 significantly increased the frequency of granzyme B (GZMB)^+^ lysosomal associated membrane protein 1 (CD107a)^+^ CD8^+^ T cells (Fig. 1H) and cellular expression of CD107a and GZMB (Fig. 1I), which was dependent on conditional 4-1BB agonist activity. In addition, GEN1046 significantly enhanced CLDN6-TCR^+^ T-cell–mediated cytotoxicity of MDA-MB-231 tumor cells more efficiently than atezolizumab or combinations of monovalent bsAb controls (Fig. 1J and K).

### GEN1046 Exhibits Antitumor Activity *In Vivo*

**Figure 2.**
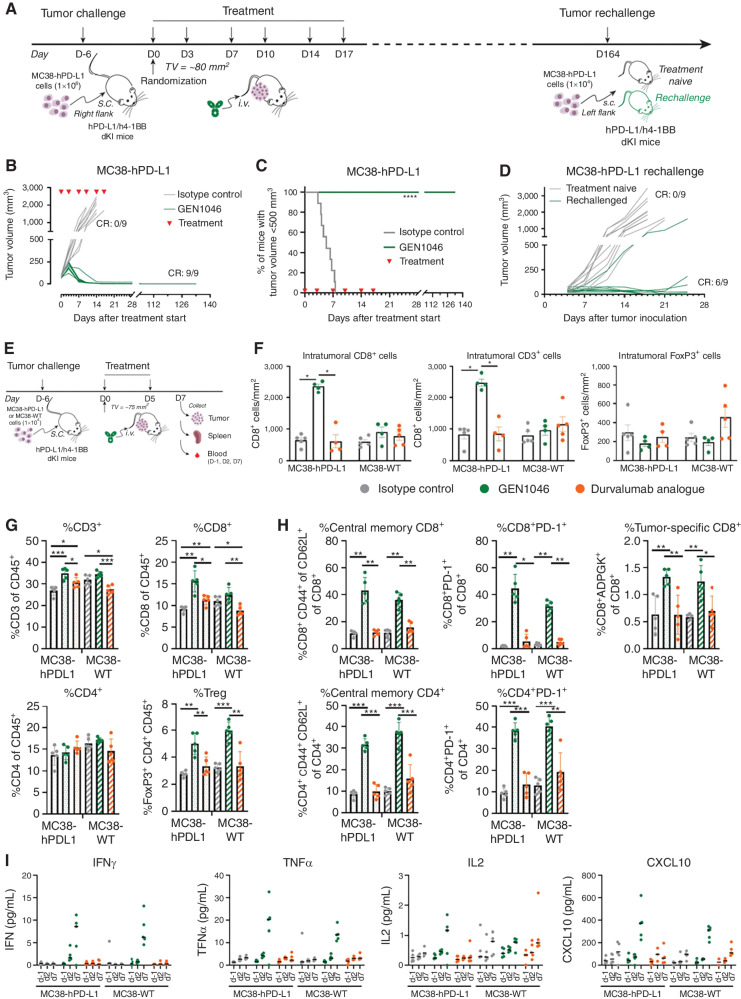
GEN1046 therapeutic efficacy and antitumor immune responses in MC38-hPD-L1 tumor–bearing hPD-L1/h4-1BB dKI mice. **A,** MC38-hPD-L1 cells (1 × 10^6^ tumor cells) were injected s.c. in the right flank of hPD-L1/h4-1BB dKI mice. After tumor establishment (average tumor volume, ∼80 mm^3^), mice were randomized and treated with GEN1046 or isotype control antibody (each 5 mg/kg intravenous) at the indicated time points (*n* = 9 per group). **B,** Tumor growth of individual mice in each group, with CR defined as number of animals with CR. **C,** Progression-free survival, defined as the percentage of mice with tumor volume smaller than 500 mm^3^, is shown as a Kaplan–Meier curve. Mantel–Cox analysis was used to compare survival between treatment groups. ****, *P* < 0.0001. **D,** Mice with CR after treatment with GEN1046 (shown in **B**) were rechallenged by s.c. injection of 1 × 10^6^ MC38-hPD-L1 tumor cells in the left flank on day 164. As a control group, a second cohort of naïve dKI animals was inoculated with 1 × 10^6^ MC38-hPD-L1 tumor cells. Tumor growth of individual mice in each group is shown. **E–I,** MC38-hPD-L1 or MC38-WT cells (1 × 10^6^ tumor cells) were injected s.c. in the right flank of hPD-L1/h4-1BB dKI mice. After tumor establishment (mean tumor volume, ∼75 mm^3^), mice were randomized and treated with GEN1046, a durvalumab analogue, or isotype control antibody (all 5 mg/kg intravenous) at the indicated time points. The mice were sacrificed 2 days after the last treatment (*n* = 5 per treatment group), and the tumors and spleens were excised. **F,** Sections of resected tumors (4 µm) were stained using anti-CD3, anti-CD8, or anti-FoxP3 antibodies by IHC. The number of positive cells was quantified per mm^2^. Mann–Whitney *U* statistical analysis was performed to compare the number of cellular subsets between treatment groups in MC38-hPD-L1 or MC38-WT tumor–bearing mice. *, *P* < 0.05. **G** and **H,** Flow cytometry analysis of dissociated splenocytes. Data from individual mice are shown as well as group mean ± SEM. *, *P* < 0.05; **, *P* < 0.01; ***, *P* < 0.001, Wilcoxon rank sum test. **I,** Peripheral blood samples were taken 1 day before treatment (d−1) and 2 days after each treatment. Cytokine analysis was performed by electrochemiluminescence immunoassay. s.c., subcutaneous; Treg, regulatory T cell.

Antitumor activity was evaluated in double knock-in (dKI) transgenic C57BL/6 mice engineered with human PD-L1 (hPD-L1) and a 4-1BB extracellular domain that were subcutaneously engrafted with MC38 tumor cells transfected with hPD-L1 (MC38-hPD-L1) to establish tumors (Fig. 2A). GEN1046 (5 mg/kg) twice weekly for three cycles induced complete tumor regression in all animals and significantly increased progression-free survival (defined as tumor size <500 mm^3^) versus controls (Fig. 2B and C). Six of nine animals with complete regression showed no tumor outgrowth upon rechallenge with MC38-hPD-L1 tumors, suggesting that GEN1046-treated animals had developed immunologic memory (Fig. 2D).

To further characterize the antitumor immune response *in vivo*, we performed immunophenotyping of tumors and spleens resected from MC38-hPD-L1 or MC38-wild-type (WT) tumor–bearing hPD-L1/h4-1BB dKI mice treated with GEN1046, a durvalumab analogue, or an isotype control antibody (5 mg/kg) twice weekly for one cycle (Fig. 2E). GEN1046 induced an increase in CD3^+^ T cells infiltrating within MC38-hPD-L1 tumors, the majority of which were CD8^+^, whereas the durvalumab analogue did not induce any changes in these T-cell subsets (Fig. 2F). In the spleen, GEN1046 induced significant increases in the percentages of CD3^+^ and CD8^+^ cells, whereas the percentage of overall CD4^+^ T cells remained unchanged (Fig. 2G). Furthermore, GEN1046 induced significant increases in the percentages of central memory and PD-1^+^ CD8^+^ and CD4^+^ T cells and tumor-specific CD8^+^ T cells compared with the isotype control and the durvalumab analogue (Fig. 2H). Whereas intratumoral FoxP3^+^ cells remained unchanged (Fig. 2F), GEN1046 significantly increased the percentage of FoxP3^+^ CD4^+^ regulatory T cells in spleen (Fig. 2G). The biological activity of GEN1046 on T cells was reflected by increases in IFNγ, TNFα, IL2, and CXCL10 in peripheral blood in MC38-hPD-L1 and MC38-WT tumor–bearing mice (Fig. 2I). The animals did not show any abnormal behavior or body weight loss during the study.

### GEN1046 Enhances Expansion of Tumor-Reactive, Tumor-Infiltrating Lymphocytes *Ex Vivo*

**Figure 3.**
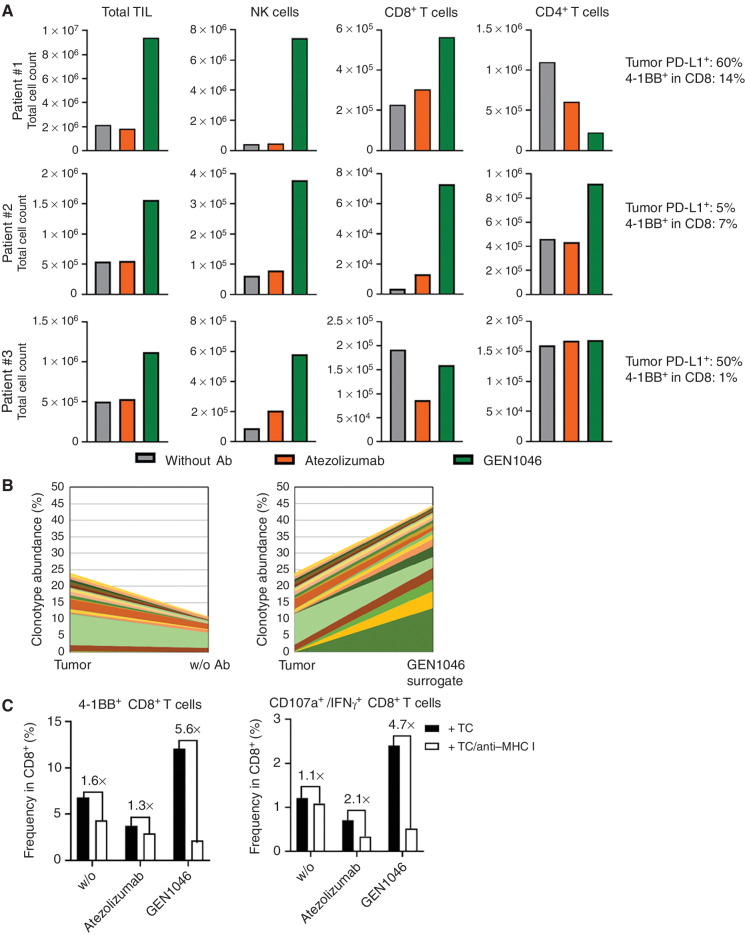
GEN1046 promotes TIL expansion from patient-derived tumor tissue. Tumor tissues resected from patients with NSCLC were cut into pieces of 1 to 2 mm^3^ and cultured in the presence of IL2 (10–50 U/mL) and GEN1046 (or a GEN1046 surrogate comprising the PD-L1–specific Fab arm of GEN1046 and a nonhumanized variant of the 4-1BB–specific Fab arm or atezolizumab (0.2 µg/mL), or with IL2 only for 14–17 days. **A,** Cell numbers after expansion were determined by flow cytometry, and total TILs, CD8^+^ T cells, CD4^+^ T cells, and NK cells are shown for three patients. Tumor PD-L1 expression and 4-1BB expression by CD8^+^ T cells in the specimen at baseline are indicated. **B,** TCR repertoire analysis was performed by *TRB* RNA sequencing of the expanded TILs and the tumor fragments. Cumulative frequency of shared clonotypes, the 20 most abundant clonotypes in the GEN1046 surrogate–treated cultures, is shown. **C,** TILs expanded as in **A** were restimulated with enzymatically digested autologous tumor (TC) in the presence or absence of an MHC I–blocking antibody. Expression of 4-1BB and intracellular expression of IFNγ and CD107a in CD8^+^ T cells were analyzed by flow cytometry.

We evaluated whether GEN1046 could improve expansion of tumor-infiltrating lymphocytes (TIL) in tumor specimens obtained from patients with non–small-cell lung cancer (NSCLC). In all three NSCLC specimens evaluated, GEN1046 increased TIL expansion versus atezolizumab in the presence of low-dose IL2 (Fig. 3A). CD8^+^ T-cell expansion increased in two specimens in which a proportion of CD8^+^ T cells expressed 4-1BB at baseline (patients 1 and 2), but not in a specimen with negligible baseline 4-1BB expression on CD8^+^ T cells (patient 3). Sequencing of the TCR β locus showed specific expansion of T-cell clonotypes in GEN1046-treated TIL cultures (Fig. 3B). Restimulation of expanded TILs with autologous tumor cells revealed that GEN1046-expanded CD8^+^ TILs contained greater proportions of cells that upregulated 4-1BB and IFNγ/CD107a upon restimulation compared with atezolizumab and IL2 alone, which was dependent on MHC I (Fig. 3C).

GEN1046 was well tolerated in cynomolgus monkeys at dose levels up to 30 mg/kg [once every 3 weeks (Q3W) for two 21-day cycles] in a pivotal good laboratory practice toxicity study. The binding affinities of GEN1046 to recombinant cynomolgus monkey PD-L1 and 4-1BB were in the subnanomolar range (*K*_D_ PD-L1: 0.27 nmol/L; 4-1BB: 0.25 nmol/L), similar to its affinities for human PD-L1 and 4-1BB. No GEN1046-related changes in aspartate transaminase (AST) or alanine transaminase (ALT) levels were observed (Supplementary Table S1).

The pharmacodynamic effects, antitumor activity, and safety profile observed in the preclinical setting demonstrated the therapeutic potential of GEN1046, thus supporting the initiation of a first-in-human, phase I study in patients with advanced solid tumors.

### First-in-Human Study Demonstrates Pharmacokinetic/Pharmacodynamic Profile Supportive of Mechanism of Action

Sixty-one patients with advanced solid tumors were enrolled in the dose-escalation part of the phase I/IIa trial (NCT03917381) and received GEN1046 at nine escalating dose levels (25–1,200 mg Q3W). The most common tumor types were colorectal (19.7%; *n* = 12), ovarian (14.8%; *n* = 9), pancreatic (9.8%; *n* = 6), and lung (NSCLC: 9.8%; *n* = 6; Table 1). Patients were heavily pretreated, having received a median (range) of three (1–11) previous anticancer treatments. Best response to last prior systemic anticancer treatment was progressive disease (PD) in 24 (39.3%) patients. Twenty-three (37.7%) patients had previously received a PD-(L)1 inhibitor; among these patients, eight (34.8%) had best response of PD on last prior PD-(L)1 inhibitor.

**Table 1.** Characteristics of patients treated with GEN1046

Characteristic	Patients (*N* = 61)^a^
Age, median (range), y	59 (23–79)
Female	28 (45.9)
Cancer type, *n*^b^	
Colorectal	12 (19.7)
Ovarian	9 (14.8)
Pancreatic	6 (9.8)
NSCLC	6 (9.8)
Other	28 (45.9)
ECOG PS	
0	34 (55.7)
1	27 (44.3)
Median prior regimens, *n* (range)	3 (1–11)
Prior treatment with a PD-(L)1 inhibitor	23 (37.7)

NOTE: Values are number (%) of patients unless specified otherwise.

Abbreviation: ECOG PS, Eastern Cooperative Oncology Group performance status.

^a^Numbers of patients assigned to each GEN1046 dose group were as follows: 25 mg, *n* = 4; 50 mg, *n* = 5; 80 mg, *n* = 9; 100 mg, *n* = 6; 140 mg, *n* = 6; 200 mg, *n* = 9; 400 mg, *n* = 9; 800 mg, *n* = 9; 1,200 mg, *n* = 4.

^b^Tumor types occurring in <5 patients are categorized as “other.”

**Figure 4.**
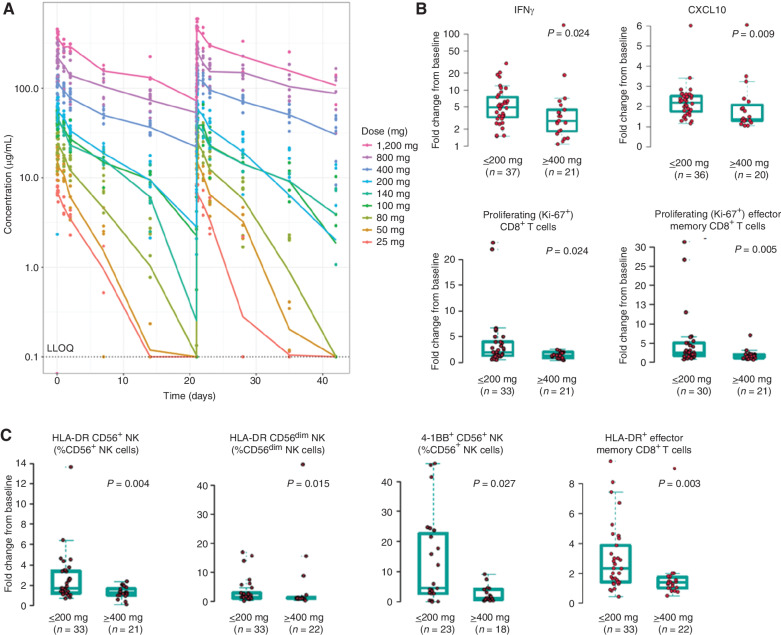
Pharmacokinetics and pharmacodynamics of GEN1046 in patients with advanced solid tumors. **A,** Mean plasma concentration of GEN1046 during the first two dosing cycles with administration Q3W. **B** and **C,** Maximal fold change from baseline in pharmacodynamic markers measured in peripheral blood during cycle 1 in patients receiving low (≤200 mg) and high (≥400 mg) doses of GEN1046. *P* values from the Wilcoxon–Mann–Whitney test. LLOQ, lower limit of quantification.

The pharmacokinetics of GEN1046 evaluated in all 61 patients indicated peak concentrations shortly after the end of infusion, with no significant accumulation at doses lower than 200 mg Q3W. The mean terminal half-life after the first dose appeared to range from 2.3 to 10.3 days across doses (Fig. 4A).

Consistent with the present study's preclinical data, treatment with GEN1046 significantly increased levels of peripheral blood pharmacodynamic markers associated with its proposed mechanism of action in patients, including IFNγ, CXCL10, proliferating (Ki-67^+^) total and effector memory CD8^+^ T-cell counts (Fig. 4B and C), and activated NK-cell counts (HLA-DR^+^ total NK cells, CD56^dim^, and 4-1BB^+^ CD56^+^ total and CD56^dim^ NK cells; Fig. 4C). Lower dose levels (≤200 mg) were associated with greater increases in peripheral immune modulation than higher dose levels (≥400 mg), concordant with the bell-shaped response curve characteristic of trimer-forming bsAbs, because monovalent saturation of the targets precludes cross-linking in conditions of agent excess (27).

### GEN1046 Demonstrates Manageable Safety Profile with Early Evidence of Antitumor Activity

GEN1046 was generally well tolerated. Dose-limiting toxicities (DLT) occurred in six (9.8%) patients (25 mg, *n* = 1; 80 mg, *n* = 1; 140 mg, *n* = 2; 200 mg, *n* = 1; 800 mg, *n* = 1) and included grade 4 febrile neutropenia (*n* = 2), grade 3 immune-mediated nephritis (*n* = 1), grade 3 ALT increase (*n* = 1), grade 3 AST/ALT increase (*n* = 1), and grade 3 transaminase elevation (*n* = 1; Supplementary Table S2). Both cases of febrile neutropenia were managed with granulocyte colony-stimulating factor, and neither event recurred upon reexposure to GEN1046. All DLTs resolved without sequelae. The MTD was not reached.

To help identify the expansion dose, a semimechanistic, physiologically based pharmacokinetic/pharmacodynamic model that incorporated dynamic simultaneous binding of GEN1046 to PD-L1 and 4-1BB in tumors and lymph nodes was used to evaluate two key surrogates of efficacy: receptor occupancy of PD-L1 in tumors and trimer formation between GEN1046, tumor cells expressing PD-L1, and immune cells expressing 4-1BB (28). This integrated model predicted a bell-shaped curve for average trimer formation that peaked near 100 mg Q3W, with doses greater than 100 mg Q3W resulting in reduced mean trimer levels. This was consistent with observed pharmacodynamic data, which showed greater immune effects at dose levels of 200 mg or lower.

Treatment-related adverse events (AE) occurred in 43 (70.5%) patients. The most common treatment-related AEs were transaminase elevations, hypothyroidism, and fatigue (Table 2). Most treatment-related AEs were grade 1–2; 17 (27.9%) patients had at least one treatment-related grade 3–4 AE. Treatment-related grade 3 transaminase elevations occurred in six (9.8%) patients; no grade 4–5 transaminase elevations occurred. Transaminase increases rapidly improved with corticosteroid administration. No treatment-related AEs of increased bilirubin were reported. Except for one grade 4 event, all occurrences of treatment-related hypothyroidism were grade 1–2. Treatment-emergent AEs (TEAE) are reported in Supplementary Table S3. Six (9.8%) patients discontinued treatment due to TEAEs (Supplementary Table S4), three of whom discontinued treatment due to grade 3 transaminase elevations (elevated ALT/AST and hepatotoxicity in one patient, elevated ALT/AST in one patient, and elevated ALT alone in one patient).

**Table 2.** Treatment-related AEs reported in >2 patients treated with GEN1046 (*n* = 61)

Event	All grades	Grade 1	Grade 2	Grade 3	Grade 4
Transaminase elevation	16 (26.2)	5 (8.2)	5 (8.2)	6 (9.8)	0
Hypothyroidism	12 (19.7)	2 (3.3)	9 (14.8)	0	1 (1.6)
Fatigue	8 (13.1)	5 (8.2)	2 (3.3)	1 (1.6)	0
Hyperthyroidism	5 (8.2)	4 (6.6)	1 (1.6)	0	
Nausea	5 (8.2)	5 (8.2)	0	0	0
Diarrhea	4 (6.6)	3 (4.9)	1 (1.6)	0	0
Amylase increased	3 (4.9)	0	3 (4.9)	0	0
Asthenia	3 (4.9)	2 (3.3)	1 (1.6)	0	0
Blood alkaline phosphatase increased	3 (4.9)	3 (4.9)	0	0	0
Gamma-glutamyl transferase increased	3 (4.9)	0	1 (1.6)	2 (3.3)	0
Lipase increased	3 (4.9)	1 (1.6)	2 (3.3)	0	0
Neutropenia	3 (4.9)	0	0	3 (4.9)	0
Rash	3 (4.9)	3 (4.9)	0	0	0

NOTE: Values are number (%) of patients.

**Figure 5.**
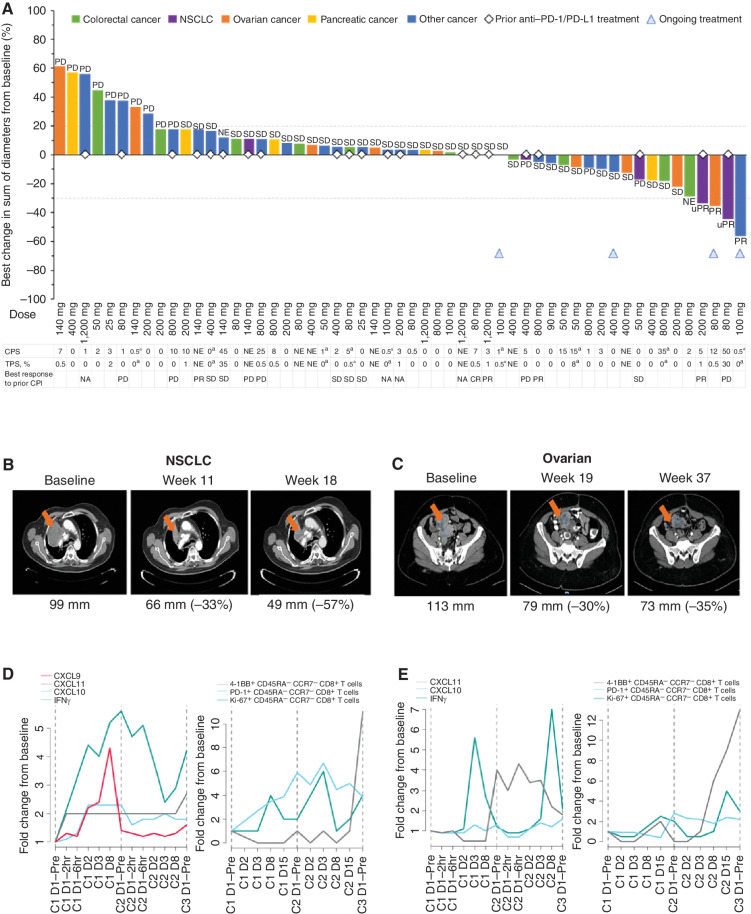
GEN1046 antitumor efficacy in patients with advanced solid tumors. **A,** Waterfall plot of best relative percent change from baseline in tumor size. **B**–**E,** CT over time (**B** and **C**) and plots of immunologic changes in the periphery (**D** and **E**) following the first dose of GEN1046 in a patient with metastatic NSCLC treated with 200 mg GEN1046 (**B** and **D**) and a patient with metastatic ovarian cancer treated with 80 mg GEN1046 (**C** and **E**). Numbers (percentages) below CT scans are sum of diameter of target lesion (percent change from baseline). NA, not applicable; NE, not evaluable. ^a^Archival formalin-fixed paraffin-embedded block of tumor tissue obtained >5 years prior was evaluated; loss of PD-L1 immunoreactivity may have occurred.

At the time of data cutoff (February 12, 2021), four (6.6%) of 61 patients remained on treatment (Supplementary Table S4). With a median (range) follow-up of 9.4 (0.3–20.0) months, disease control [defined as stable disease (SD) or better per Response Evaluation Criteria in Solid Tumors (RECIST) version 1.1; ref. 29) occurred in 40 of 61 patients (65.6%; 95% confidence interval, 52.3–77.3; Fig. 5A). Four patients achieved a partial response (PR), including confirmed PR in one patient with triple-negative breast cancer (100-mg dose level) and confirmed PR in one patient with ovarian cancer (80 mg), neither of whom had received prior anti–PD-(L)1 therapy. Unconfirmed PRs (uPR) were reported in two patients with NSCLC (80 and 200 mg), both of whom had previously progressed on anti−PD-(L)1 therapy. Thirty-six (59.0%) patients had SD as best response, all of whom had SD at first response assessment at week 6 (± 1 week; Supplementary Fig. S3).

Among the six patients with NSCLC (GEN1046 dose levels: 50–400 mg), two patients (80 and 200 mg) achieved uPR, two (80 and 140 mg) had SD, and two (50 and 400 mg) experienced PD. All six patients had previously been treated with a CPI. Best response to previous CPI treatment was PD (*n* = 3), SD (*n* = 1), PR (*n* = 1), and complete response (CR; *n* = 1), with a range of 43 to 1,486 days since last CPI treatment. Based on evidence from the pharmacokinetic/pharmacodynamic model and observed clinical safety, efficacy, and pharmacodynamic data from the dose-escalation study, 100 mg Q3W was chosen as the expansion dose of GEN1046.

Representative computed tomography (CT) scans of the patient with NSCLC who had an uPR are presented in Fig. 5B. This patient was a 67-year-old man with stage IV *KRAS^+^* adenocarcinoma who had received five prior lines of therapy in the metastatic setting. His most recent prior treatment had been bevacizumab in combination with an investigational drug, which led to best response of SD. The patient began receiving GEN1046 200 mg Q3W approximately 2 months following last therapy and experienced PR at week 11, with further reduction in target lesion size measured at week 18 despite detection of a new brain lesion at week 14. Due to the good systemic disease control and clinical benefit experienced by the patient, GEN1046 was continued beyond progression until week 31, when the patient again experienced progression (target lesion in the lung) and discontinued treatment.

Representative CT scans of the patient with ovarian cancer who had a confirmed PR are presented in Fig. 5C. This 55-year-old woman with stage IV granulose ovarian cancer with no known driver mutations had received five prior lines of therapy in the metastatic setting, most recently bevacizumab, which led to a best response of PD. The patient began GEN1046 (80 mg Q3W) approximately 16 months following last therapy and achieved PR at week 19, which was maintained until week 60 (when SD was observed). The patient remains on treatment, having received 30 cycles of GEN1046 as of August 2021.

Exploratory pharmacodynamic analyses of these two case studies showed induction of peripheral IFNγ and IFN-responsive chemokines (CXCL9–11) and increases in proliferating total and PD-1^+^ CD8^+^ effector memory T-cell counts following the first dose of GEN1046 (Fig. 5D and E). Based on analyses of biopsy samples acquired before treatment with GEN1046, the PD-L1 tumor proportion scores (TPS) were 1% in the patient with NSCLC and 0.5% in the patient with ovarian cancer; the PD-L1 combined positive scores (CPS) were 5 and 12; 4-1BB was expressed in 147 and 26 cells/mm^2^; and the densities of CD8^+^ T cells in the TME were 385 and 518 cells/mm^2^, respectively (Supplementary Fig. S4A and S4B). Among 52 patients with evaluable tumor biopsy samples, 84.6% harbored PD-L1^−^ tumors per TPS (*n/N* = 44/52), and 50.0% of those patients were also PD-L1^−^ per CPS (*n/N* = 22/44). The median PD-L1 TPS and CPS were 0% and 1, respectively, and the 4-1BB and CD8 median values were 23 and 120 cells/mm^2^, respectively.

## DISCUSSION

Using bsAbs to target a combination of immunologic pathways provides a promising approach to improve the clinical benefit of PD-1/PD-L1 blockade. GEN1046 is an investigational first-in-class, bispecific immunotherapy designed to enhance anti–PD-L1–mediated antitumor activity by further reinvigorating the immune response via conditional agonist 4-1BB activation. The preclinical studies described here clearly demonstrate that GEN1046 exhibits conditional 4-1BB agonist activity dependent on cross-linking with PD-L1 while retaining the ability to block PD-L1/PD-1 independently of 4-1BB binding. Through this dual mechanism of action, GEN1046 promotes and strengthens interactions between DCs and T cells, enhances activation, proliferation, and effector functions of human T cells *in vitro*, promotes expansion of tumor-reactive TILs *ex vivo*, and induces antitumor activity in tumor-bearing mice *in vivo*.

Within the TME, 4-1BB is expressed by a subset of tumor-infiltrating CD8^+^ T cells characterized by coexpression of multiple TCR-inducible molecules, including high levels of PD-1 (16, 30). The TCR repertoire analyses suggested that 4-1BB^+^ intratumoral CD8^+^ T cells are enriched for tumor antigen–specific T cells, as confirmed by functional assays showing that 4-1BB^+^ CD8^+^ TILs exhibit autologous tumor-cell reactivity (12, 16, 17). This aligns with the observation that a high frequency of intratumoral PD-1^+^ 4-1BB^+^ CD8^+^ T cells positively correlates with clinical response to pembrolizumab (31). PD-L1 expression is upregulated by tumor cells and tumor-infiltrating myeloid cells (32) or DCs (33), providing a potential source of PD-L1 required for GEN1046 conditional stimulation of 4-1BB. GEN1046 enhanced expansion of TILs from patient-derived PD-L1^+^ tumor tissue, and the level of 4-1BB expression on CD8^+^ T cells before culture with GEN1046 was associated with expansion of this subpopulation in TILs. Previous studies have shown preferential augmentation of cytotoxicity by intratumoral 4-1BB–expressing tissue-resident CD8^+^ T cells expressing CD103 upon treatment with a combination of anti–PD-L1 and anti–4-1BB mAbs (34, 35). Our data suggest that target cross-linking may directly mediate antigen-specific T-cell–mediated cytotoxicity of PD-L1^+^ tumor cells. Furthermore, expression of PD-1 and 4-1BB on CD4^+^ and CD8^+^ T cells and PD-L1 on DCs has been reported in the tumor-draining lymph nodes from patients (36), suggesting that GEN1046 may further boost tumor-specific T-cell activation in tumor-draining lymph nodes.

GEN1046-mediated T-cell stimulation was consistently superior to PD-1/PD-L1 blockade alone across a variety of *in vitro* assays, suggesting that dual targeting of 4-1BB and PD-L1 may improve antitumor activity *in vivo*. These findings align with previously published data on PD-L1×4-1BB bsAbs (37–41). Here we showed that GEN1046 induced potent antitumor activity in MC38-hPD-L1 tumor–bearing mice and conferred protection against secondary tumor challenge. GEN1046 significantly enhanced intratumoral CD8^+^ T-cell counts in MC38-hPD-L1 tumor–bearing mice, a finding that can be attributed to the combination of PD-L1 blockade with 4-1BB agonist activity, as PD-L1 blockade alone with a durvalumab analogue had no such effect. The knock-in mouse model represents the closest possible approach to clinical reality. Although hPD-L1 expression within the tumor was required for intratumoral CD8^+^ T-cell accumulation, GEN1046 treatment also increased the frequencies of splenic tumor-specific and memory phenotype T cells and proinflammatory and T-cell chemoattractant cytokines in MC38-WT tumor–bearing mice, suggesting that GEN1046 may leverage lymphoid tissue-resident PD-L1–expressing cells to costimulate T cells.

The mechanism of action of GEN1046 is supported by the peripheral pharmacodynamic changes observed in patients in the phase I trial. Consistent with preclinical data, treatment with GEN1046 significantly increased levels of biomarkers such as IFNγ, CXCL10, and proliferating (Ki-67^+^) total and effector memory CD8^+^ T-cell counts. Elevations in Ki-67^+^ CD8^+^ T cells and other biomarkers have been correlated with improved clinical response to PD-(L)1–targeted mAbs (42–44). The magnitude of enhancements in T-cell and NK-cell function was generally greater than that observed with PD-(L)1– and 4-1BB–targeted mAbs alone (23, 43–45). With utomilumab, a weak 4-1BB agonist, T-cell and NK-cell elevations did not significantly increase with dose (23). Although urelumab induced expression of a range of IFN-induced cytokines in patients with cancer (22), most increases were of a substantially lower magnitude than those observed with GEN1046 in this study and had different kinetics (22). These observations suggest an enhanced immune response with GEN1046 compared with PD-(L)1– and 4-1BB–targeted agents individually.

GEN1046 showed a manageable safety profile in the dose-escalation study in 61 heavily pretreated patients with advanced solid tumors. Febrile neutropenia, which was observed as a DLT in two of six patients with DLTs, had been previously reported in patients treated with urelumab (22). Neutropenia could be related to the fact that neutrophils reportedly express CD137 (46) and to inflammatory cytokines mediating myelosuppression (47). Most treatment-related AEs with GEN1046 were grade 1–2 regardless of dose increases. Treatment-related grade 3 transaminase elevations occurred in 9.8% of patients and were managed with corticosteroid treatment. No treatment-related increases in bilirubin or grade 4 increases in liver transaminases were reported in this series of patients, and no treatment-related deaths occurred. Three (4.9%) patients discontinued GEN1046 due to transaminase elevations. This safety profile compares favorably with that of urelumab, which was associated with higher rates of grade 3–4 treatment-related transaminase elevations (13.5%–16.6%), discontinuations due to treatment-related AEs (16%), and two cases of fatal hepatotoxicity at doses associated with antitumor activity (≥1 mg/kg; ref. 22). The comparatively favorable safety profile of GEN1046 may be mediated by the conditional nature of its 4-1BB agonist activity, which likely limits widespread T-cell–mediated cytotoxicity by requiring engagement of PD-L1 before 4-1BB, rather than allowing activation of 4-1BB alone. We have observed that parental anti–4-1BB mouse mAb in a bivalent format induced elevated transaminase levels and increased intrahepatic CD8^+^ T-cell numbers in a manner that was further aggravated by combination treatment with a PD-L1 mAb (48). By contrast, animals treated with a mouse PD-L1×4-1BB bsAb did not manifest signs of hepatic inflammation, such as elevated serum transaminase activity levels or CD8^+^ T-cell infiltration of the liver, at dose levels that provided effective antitumor activity *in vivo*. Given the overlap of AEs (hepatotoxicity and neutropenia) with GEN1046 and other cancer agents, careful safety monitoring in future combination studies will be needed.

In this population of patients with advanced disease who had received multiple prior lines of therapy, GEN1046 demonstrated encouraging single-agent activity, with 65.6% of patients experiencing disease control, including uPRs in two patients with NSCLC and confirmed PRs in one patient with ovarian cancer and one patient with triple-negative breast cancer. Although a majority of outcomes to GEN1046 were SD as best response, the high disease control rate suggests clinical significance for this population of heavily pretreated patients (median, three prior regimens) with limited response to their last line of treatment (39.3% had best response of PD on last treatment). Of note, patients were not preselected based on PD-L1 status, and a high fraction of patients had PD-L1^−^ tumors (84.6% PD-L1^−^ by TPS; 50% of those also PD-L1^−^ by CPS). Clinical benefit was observed across doses of GEN1046 and in different tumor types, including those resistant to prior immunotherapy and in diseases typically less sensitive to CPIs, indicating that activity of GEN1046 is at least partly related to 4-1BB costimulation enabled by PD-L1 blockade. Responses occurred in patients with NSCLC who had received prior PD-(L)1 therapy, as well as in a patient with triple-negative breast cancer and a patient with ovarian cancer, neither of whom had received prior anti−PD-(L)1 treatment.

The MTD for GEN1046 was not reached after evaluation of dose levels up to 1,200 mg Q3W. The 100-mg Q3W dose level was chosen as the expansion dose for GEN1046 because this regimen was predicted to balance optimal levels of GEN1046:PD-L1:4-1BB trimers with PD-L1 receptor occupancy in pharmacokinetic/pharmacodynamic modeling and was associated with a manageable safety profile and antitumor activity in the phase I study.

Importantly, the complementary, bispecific action of GEN1046 was associated with antitumor responses in TMEs expressing both targets. In the presented NSCLC case study, the patient's tumor profile was characterized as low PD-L1^+^ (TPS: 1%) and 4-1BB^+^ (147 cells/mm^2^). The ovarian cancer case exhibited PD-L1 expression (CPS: 12), with lower 4-1BB cell density (26 cells/mm^2^) and marked infiltration of CD8^+^ T cells (518 cells/mm^2^). Although GEN1046 may exert its activity in the TME, it is likely that immune activation also occurs in the tumor-draining lymph nodes, where both targets are expected to be present.

Based on its acceptable safety profile, GEN1046 appears to circumvent the key safety limitation of hepatotoxicity associated with 4-1BB mAbs. GEN1046 conferred clinical benefit in patients with different tumor types, including those with disease less sensitive to CPIs. Our preclinical and limited clinical experience thus far suggests that reinvigoration of the TME through immune modulation and mobilization of antitumor immunity through enhanced proliferation, activation, and effector function of T cells can be achieved to potentially offer an improved approach to immunotherapy. Although quantifiable immunologic changes were observed in the periphery, formal biomarker analyses are needed to guide accurate identification of patients most likely to derive clinical benefit from GEN1046. The ongoing phase II part of this study will provide additional efficacy and safety data, as well as exploratory analyses of potential biomarkers of response, that will help define the most suitable malignancies and combination strategies.

## METHODS

### Preclinical

#### Antibodies.

Commercially available and research antibodies are listed in Supplementary Methods Tables S1 and S2. The mAbs were recombinantly produced in a human IgG1 backbone using previously described methods (49). The mutations *L234F*, *L235E*, and *D265A* were introduced to abrogate Fc binding to IgG Fcγ receptors and C1q (50). The F405L and K409R mutations were introduced to allow generation of bsAbs by controlled Fab-arm exchange (24, 25). The HIV-1 gp120-specific isotype control antibody based on IgG1-b12 (51) was used to generate bsAbs with one nonbinding Fab arm (e.g., bsAb-ctrlx4-1BB). The procedure for the generation and selection of mAb-PD-L1, mAb-4-1BB, and GEN1046 is described in the Supplementary Methods.

#### Cell lines.

MDA-MB-231 cells (ATCC, HTB-26; RRID:CVCL_0062) were cultured in Dulbecco-modified Eagle medium (DMEM) with 10% FBS. MDA-MB-231 hCLDN6 cells were generated by lentiviral transduction of MDA-MB-231 cells with human CLDN6. K562 cells (ATCC, CCL-243; RRID:CVCL_0004) were cultured in Iscove-modified Dulbecco medium (IMDM) with 10% FBS. K562-h4-1BB and K562-hPD-L1 cells were generated by retroviral transduction. ES-2 cells (ATCC, CRL-1978; RRID:CVCL_3509) were cultured in IMDM with 10% FBS. Cell lines were not passaged more than 20 times before use in experiments. Cell-line authentication and *Mycoplasma* testing were conducted by Eurofins PCR testing service. Cell culture and media buffers are listed in Supplementary Methods Table S3.

For initiation of the MC38 tumor model in hPD-L1/h4-1BB dKI mice, MC38 cells (WT or expressing hPD-L1; MC38-hPD-L1; developed by Nanjing Galaxy Biopharma, and licensed to Crown Bioscience) were cultured in DMEM supplemented with 10% heat-inactivated FBS and hygromycin B 50 µg/mL (Invitrogen; 10687010; 37°C; 5% CO_2_). Cells were generally cultured for 2 weeks, passaged three to four times, and were in the exponential growth phase when harvested. Cell batches with greater than 95% viability (trypan blue staining) were injected for tumor inoculation. Cell line authentication was performed by single-nucleotide polymorphism assays, and *Mycoplasma* testing was conducted before banking (Crown Bioscience).

#### Mice and tumor models.

*C57BL/6-Cd274^tm1 (hCD274)^ Cd137^tm1 (hCD137)^/Bcgen* mice engineered to express human PD-L1 and the 4-1BB extracellular domain in the mouse PD-L1 and 4-1BB gene loci (hPD-L1/h4-1BB dKI mice) were purchased from Beijing Biocytogen. The hPD-L1 and 4-1BB extracellular domain were confirmed to be inducibly expressed on splenic CD8^+^ T cells. In all experiments, age-matched (9–12 weeks) female animals were used. All animal studies were approved by local regulatory authorities for animal welfare. All mice were kept in accordance with federal and state policies on animal research at Crown Bioscience.

Mice were subcutaneously injected in the flank with 10^6^ MC38-WT or MC38-hPD-L1 cells in 100-µL PBS. When tumors reached a mean volume of 75 to 80 mm^3^, mice were randomized to receive intravenous injection of GEN1046 5 mg/kg or control antibodies in PBS. Tumor growth was evaluated twice weekly by caliper measurements. Resected tumors and spleens as well as peripheral blood samples were analyzed by IHC, flow cytometry, and cytokine analysis, respectively.

#### Cell isolation and enrichment.

The PBMCs were isolated from buffy coats of healthy human blood donors (Transfusionszentrale, University Hospital, Mainz, Germany) by density centrifugation over a Ficoll–Paque gradient (VWR; 17-5446-02).

CD14^+^ monocytes were enriched from buffy coats by magnetic-activated cell sorting using CD14 magnetic microbeads (Miltenyi, 130-050-201) and an AutoMACS Pro Separator (Miltenyi, 130-092-545). CD8^+^ T-cell populations were enriched from the CD14^−^ fraction or whole PBMCs by magnetic-activated cell sorting using CD8 microbeads (Miltenyi, 130-045-201).

Spleens were mechanically dissociated to single-cell suspension using a 70-µm strainer, and, after centrifugation, erythrocytes were lysed with RBC Lysis Buffer (BioGems; 64010-00-100).

#### Differentiation of iDCs.

CD14^+^ monocytes were incubated with 100 to 200 ng/mL granulocyte–macrophage colony-stimulating factor (BioLegend; 766106) and 50 to 200 ng/mL IL4 (BioLegend; 766206) in DC medium (Supplementary Methods). Fresh medium supplemented with granulocyte–macrophage colony-stimulating factor and IL4 was supplied as appropriate. Nonadherent and adherent cells were harvested between days 5 and 7, pooled, and collectively referred to as iDCs.

#### Electroporation.

iDCs or CD8^+^ T cells were suspended at 20 × 10^6^ or 60 × 10^6^ cells/mL, respectively, in X-Vivo15 medium. For each electroporation, a 250-µL cell suspension was transferred to electroporation cuvettes (4.0-mm gap size, VWR, 732-0023) and kept at room temperature (iDCs) or on ice (T cells). RNA encoding CLDN6 (0.3–3 µg) was electroporated into iDCs using an ECM 830 Electroporation System (BTX, 45-0002). RNA encoding the α and β chains of CLDN6-TCR (10 µg each) with or without RNA encoding PD-1 (0.4–10 µg) was electroporated into T cells using the same device. Expression of electroporated proteins was confirmed by flow cytometry.

#### Imaging of DC–T-cell interaction.

DCs were seeded on poly-L-lysine (0.1 µg/mL; 2 × 10^6^ cells/well) coated coverslips for 4 hours, cocultured with CD3/CD28 bead–stimulated (Thermo Fisher Scientific, 11132D) activated CD8^+^ T cells (4 × 10^6^ cells/well) in the presence of 0.125 to 0.25 µg/mL GEN1046 or control antibodies and analyzed by immunofluorescence or live-cell imaging.

#### Reporter assays.

The effect of GEN1046 on the functional interaction of PD-1 with PD-L1 was determined using the PD-1/PD-L1 Blockade Bioassay (Promega, J1255). Briefly, CHO-K1 cells expressing human PD-L1 and an engineered cell-surface protein designed to activate TCRs in an antigen-independent manner were cocultured for 6 hours with Jurkat T cells expressing human PD-1 and a luciferase reporter driven by a nuclear factor of activated T cells (NFAT) response element in the presence of GEN1046 or control antibodies. Luciferase reporter activity was measured by luminescence using an Infinite F200 Pro plate reader (Tecan Life Sciences) or a multilabel plate reader (EnVision, PerkinElmer). 4-1BB agonist activity was evaluated using human 4-1BB effector cells (T&U GloResponse NFκB-luc2/4-1BB Jurkat cells; Promega, CS196003) and the BioGlo Luciferase Assay (Promega, G720A). The 4-1BB effector cells were cocultured for 6 hours with PD-L1^+^ ES-2 cells or cultured without ES-2 cells in the presence of GEN1046 or control antibodies. Luminescence was recorded as above.

#### T-cell proliferation assays.

PBMCs were labeled with carboxyfluorescein succinimidyl ester (CFSE) using the Vybrant CFDA SE Cell tracer kit (Life Technologies; V12883) and stimulated with anti-CD3 antibody in T-cell assay media at 7.5 × 10^4^ cells/well. Cells were cultured in the presence of GEN1046 or control antibodies for 4 days, and CFSE dilution was analyzed by flow cytometry. Supernatants were harvested for cytokine analysis 48 hours after the start of culture.

CD8^+^ T cells were isolated from healthy donor PBMCs (HLA-A*02) and electroporated with RNA encoding an HLA-A*02–restricted, CLDN6-TCR (and RNA encoding PD-1 for some experiments). CD8^+^ T cells were labeled with CFSE, as described above, and cocultured with CLDN6-electroporated autologous iDCs (5 × 10^4^ T cells and 5 × 10^3^ iDCs/well). Cells were treated with GEN1046 or control antibodies for 4 days. Supernatants were harvested for cytokine analysis, and cells were analyzed for T-cell proliferation by flow cytometry.

#### Cytotoxicity assay.

Electroporated CLDN6-TCR^+^ CD8^+^ T cells (HLA-A*02) were preactivated for 24 hours by coculture with CLDN6-transgenic MDA-MB-231 tumor cells (MDA-MB-231 hCLDN6) endogenously expressing PD-L1. Preactivated CD8^+^ T cells were transferred to MDA-MB-231 hCLDN6 cells that had been seeded (2 × 10^4^ cells/well) on the previous day in an xCELLigence E-plate (1 × 10^5^ T cells/well; Agilent). Cells were incubated with GEN1046 or control antibodies (0.2 µg/mL) for 120 hours with impedance measured at 2-hour intervals in an xCELLigence Real-Time Cell Analysis Instrument (Agilent). Impedance measurement data were normalized to the time of coculture start for each treatment condition, and data were expressed relative to tumor cells cultured alone (set to 100%). After 48 hours of incubation, supernatants were removed from replicate wells not subjected to impedance measurement for analysis of intracellular GZMB and CD107a. Fresh media containing Brefeldin A (BD Biosciences; 555029) were added to the cells, followed by incubation for 4 hours and flow cytometry.

#### TIL assay.

Fresh tumor resections obtained from patients with NSCLC were dissected into fragments and cryopreserved in 10% DMSO in FBS. Thawed tumor fragments were further dissected into 1 to 2 mm^3^ pieces and used for TIL expansion. Fresh or thawed pieces were transferred to a 24-well plate (VWR, 734-1604) at two pieces/well in 1-mL TIL medium (Supplementary Methods) and incubated with GEN1046 or control antibodies in the presence of 10 to 50 U/mL IL2. After 10 to 15 days, TILs were pooled by treatment condition and subjected to TCR sequencing or analyzed by flow cytometry. For TIL restimulation, single-cell suspensions were generated from freshly thawed tumor fragments by mechanical dissociation in the presence of DNase I. Cells were then cocultured with expanded TILs in a 1:1 ratio overnight with or without 10 µg/mL of an MHC I–blocking mAb (BioLegend, 311428; RRID:AB_2561492). Anti-CD107a antibody (BioLegend, 328611; RRID:AB_1227507) was added to cocultures destined for intracellular analysis at the start of restimulation; brefeldin A was added during the last 4 to 6 hours. Analysis was performed by flow cytometry.

Methods for the toxicity study in cynomolgus monkeys are provided in the Supplementary Methods.

### Clinical

#### Study design.

The dose-escalation part of the multicenter, open-label phase I/IIa study (NCT03917381) was conducted at seven sites in the United States, Spain, and Israel. Patients received single intravenous infusions of GEN1046 at flat doses ranging from 25 to 1,200 mg Q3W (21-day cycles) until PD, unacceptable toxicity, or another discontinuation criterion was met. The starting dose was selected using the no-observed-adverse-effect-level (NOAEL)–based approach with data from the toxicity study in cynomolgus monkeys in which the NOAEL dose was determined to be 30 mg/kg, as described in the Supplementary Methods. Dose escalation began with an accelerated phase consisting of single-patient cohorts followed by larger cohorts informed by a modified continual reassessment method (52) and an escalation with overdose-control design (53). During the single-patient cohort phase, the next dose level was initiated only after assessment of the 21-day DLT evaluation period for the previous dose level. Single-patient cohorts were expanded to three-patient cohorts upon occurrence of DLTs or recommendation of the safety committee. Dose reductions or interruptions of GEN1046 treatment were allowed as specified in the Supplementary Methods.

This study is being conducted in compliance with the Declaration of Helsinki, the International Council for Harmonisation Guidelines for Good Clinical Practice, and applicable local regulatory requirements. The protocol was approved by the independent ethics committee or institutional review board at each site. All patients provided written informed consent before participation.

#### Eligibility criteria.

The study enrolled patients ages ≥18 years with a histologically or cytologically confirmed, metastatic, or unresectable non–central nervous system solid tumor for whom standard therapy was not available, the patient was ineligible for, or standard therapy was unlikely to confer clinical benefit. Additional inclusion criteria included measurable disease per RECIST v1.1 (29); Eastern Cooperative Oncology Group performance status 0 to 1; and adequate renal, liver, and hematologic function. Patients were required to provide an archival or fresh tumor tissue sample obtained prior to treatment on day 1 of cycle 1. Patients were excluded if they had uncontrolled intercurrent illness; a history of intracerebral arteriovenous malformation, cerebral aneurysm, progressive brain metastases, spinal cord compression (from disease), or stroke; radiotherapy or immunosuppressive doses of corticosteroids within 14 days of first dose of GEN1046; treatment with an anticancer agent within 28 days of first dose; and toxicities from prior anticancer therapies that had not resolved to baseline levels or grade ≤1 (except alopecia, anorexia, vitiligo, fatigue, hyperthyroidism, hypothyroidism, and peripheral neuropathy).

#### Outcomes.

Primary objectives of the dose escalation were to determine the MTD and/or the recommended phase II dose and to establish the safety profile of GEN1046. Secondary objectives included pharmacokinetics and antitumor activity per RECIST v1.1. Pharmacodynamic assessments were exploratory.

AEs were coded to Medical Dictionary for Regulatory Activities v23.0 and graded per Common Terminology Criteria for Adverse Events v5.0. Venous blood samples for measurement of plasma concentrations of GEN1046 were collected before infusion, at the end of infusion, and 2 and 4 hours after infusion on day 1, before infusion on days 2 to 5, 8, and 15 of cycles 1 to 2; before and 2 hours after infusion on day 1 of cycles 3, 5, and 7 and every fourth cycle thereafter; and at treatment discontinuation. Antitumor activity was assessed by CT or MRI every 6 weeks for 50 weeks and every 12 weeks thereafter until investigator-assessed PD (unless the investigator elected to continue GEN1046 and follow modified RECIST v1.1 for immune-based therapeutics; ref. 54), start of new anticancer therapy, study withdrawal, or death, whichever occurred first. For exploratory biomarker analyses, expression of PD-L1, 4-1BB, and other cell-surface proteins was evaluated in tumor samples by IHC. Exploratory pharmacodynamic analyses in peripheral blood included measurements of key cytokines and chemokine levels and immunophenotyping. Blood samples were collected from patients at baseline and during cycles 1 and 2 [days 1 (2 and 4–6 hours postadministration), 2, 3, 8, and 15]. Serum levels of immune mediators were measured by a Meso Scale Diagnostics (MSD) Discovery multiplex immunoassay (K15209G). Cytokines and chemokines were evaluated using the V-PLEX Plus Human Biomarker 40-Plex Kit (MSD, K15209G-1) on a Meso Sector S600 instrument (MSD, IC0AA-0). Immunophenotyping of peripheral blood was conducted in whole blood collected in EDTA tubes. After lysis of red blood cells, samples were stained with antibodies for analysis on a BD FACSCanto II flow cytometer (Becton Dickinson) within 1 hour (Supplementary Methods).

#### Statistical analysis.

All analyses were performed on the full analysis set, which included all patients who received at least one dose of GEN1046. For dose escalation by the modified continual reassessment method, the relationship between DLT probability and the GEN1046 dose level was described by a Bayesian logistic regression model. After each cohort, model parameters were updated based on available DLT information. A next dose was suggested based on the posterior probability of a DLT at each dose. The MTD was to be declared when at least nine patients were evaluated at a particular dose level and the Bayesian logistic regression model recommended allocating an additional cohort to the same dose level.

### Data Availability

The preclinical data sets generated and/or analyzed during the current study are not publicly available but are available from the corresponding author upon reasonable request. Clinical trial data can be requested by qualified researchers for use in rigorous, independent scientific research as long as the trials are not part of an ongoing or planned regulatory submission. Sharing of data is subject to protection of patient privacy and respect for the patient's informed consent. The data will be provided following review and approval of a research proposal and Statistical Analysis Plan and execution of a Data Sharing Agreement. For approved requests, the data will be accessible for 12 months, with possible extensions considered. For more information on the process or to submit a request, contact clinicaltrials@genmab.com.

## Supplementary Material

Supplementary Figure
