# Supplementary material for: Preclinical Characterization and Phase I Trial Results of a Bispecific Antibody Targeting PD-L1 and 4-1BB (GEN1046) in Patients with Advanced Refractory Solid Tumors
Source: Cancer Discov. 2022 Feb 17;12(5):1248–65. doi: 10.1158/2159-8290.CD-21-1345 (PMC9662884; doi:10.1158/2159-8290.CD-21-1345)
Supplement: Supplementary Figure [file cd-21-1345_supplementary_figures_tables_and_methods_suppsm1.pdf]

Supplementary Data and Methods

Preclinical Characterization and Phase I Trial Results of a Bispecific Antibody Targeting PD-L1 and 4-1BB (GEN1046) in Patients With Advanced Refractory Solid Tumors

Supplementary Table 1. Serum transaminase levels in cynomolgus monkeys treated with GEN1046[a]

Group mean AST (U/L)						
Dose (mg/kg)		Predose	Day 1	Day 8	Day 22	Day 29
0	M	41.27 ± 5.516	45.72 ± 10.134	41.41 ± 6.981	40.19 ± 9.092	46.45 ± 4.432
	F	44.82 ± 10.032	43.16 ± 3.687	41.27 ± 10.089	34.44 ± 2.922	38.77 ± 5.177
1	M	38.28 ± 6.727	44.78 ± 6.142	41.43 ± 6.131	40.44 ± 4.841	45.23 ± 6.589
	F	67.84 ± 42.192	41.27 ± 4.555	37.82 ± 2.584	37.03 ± 4.081	43.90 ± 5.577
5	M	40.10 ± 9.845	37.90 ± 8.859	36.65 ± 7.569	33.89 ± 5.855	37.59 ± 9.052
	F	39.63 ± 39.63	36.11 ± 6.142	36.28 ± 9.948	34.59 ± 7.092	37.70 ± 7.690
30	M	42.00 ± 7.039	41.60 ± 5.051	38.69 ± 6.261	35.73 ± 3.837	37.96 ± 6.934
	F	39.40	35.81	38.39	34.99	40.26
**Group mean ALT (U/L)**						
**Dose (mg/kg)**		**Predose**	**Day 1**	**Day 8**	**Day 22**	**Day 29**
0	M	58.64 ± 19.330	71.33 ± 33.843	63.18 ± 22.491	66.53 ± 32.856	68.16 ± 31.055
	F	52.52 ± 17.393	47.03 ± 11.673	49.79 ± 16.799	41.21 ± 12.848	49.98 ± 7.517
1	M	57.50 ± 12.764	61.37 ± 19.307	62.80 ± 18.026	58.66 ± 14.204	58.90 ± 11.108
	F	62.56 ± 14.113	56.87 ± 10.426	59.05 ± 17.078	55.62 ± 20.916	66.67 ± 8.983
5	M	46.16 ± 10.790	42.93 ± 14.174	44.00 ± 15.713	39.00 ± 7.233	38.58 ± 6.703
	F	55.59 ± 35.675	44.15 ± 26.192	47.66 ± 29.448	39.52 ± 22.042	45.70 ± 25.757
30	M	43.57 ± 6.101	41.77 ± 7.141	45.66 ± 6.720	41.08 ± 10.757	40.47 ± 7.195
	F	46.71 ± 14.424	42.77 ± 7.078	50.62 ± 15.342	50.55 ± 25.532	56.42 ± 30.644

NOTE: Cynomolgus monkeys were treated with GEN1046 0, 1, 5 or 30 mg/kg intravenously (one dose Q3W for 2 cycles). Data shown are mean ± SD. AST and ALT concentrations (U/L) per group (5 animals/sex/dose level) in peripheral blood predose and at days 1, 8, 22, and 29.

Abbreviations: ALT, alanine transaminase; AST, aspartate transaminase; F, female; M, male.

[a]The binding affinity of GEN1046 to recombinant cynomolgus monkey PD-L1 and 4-1BB was in the subnanomolar range, which was similar to the binding affinity for hPD-L1 and 4-1BB (data not shown).

Supplementary Table 2. Summary of DLTs

GEN1046 dose (mg)	Patient with DLT, n	Event, grade[a]	Event duration, d	Effect on GEN1046 administration	Event outcome
25 (*n* = 4)	1	Grade 4 febrile neutropenia	3	No dose change	Resolved
50 (*n* = 5)	0	—			
80 (*n* = 9)	1	Grade 4 febrile neutropenia	6	Dose delay	Resolved
100 (*n* = 6)	0	—			
140 (*n* = 6)	2	Grade 3 immune-mediated nephritis	6	Discontinued	Resolved
		Grade 3 ALT increase	1	Dose delay[b]	Resolved
200 (*n* = 9)	1	Grade 3 AST/ALT increase	7	Discontinued[b]	Resolved
400 (*n* = 9)	0	—			
800 (*n* = 9)	1	Grade 3 transaminase elevation	3	No dose change	Resolved
1200 (*n* = 4)	0	—			

Abbreviations: ALT, alanine aminotransferase; AST, aspartate aminotransferase.

[a]Highest grade of event recorded.

[b]Per protocol, treatment with GEN1046 was interrupted in patients who experienced grade 3 ALT or AST elevations; if the event resolved to grade ≤1 within 14 days, GEN1046 may have been continued. Patients who experienced grade 4 transaminase elevations were required to permanently discontinue GEN1046.

Supplementary Table 3. TEAEs reported in >10% of patients (*n* = 61)

TEAE	All grades	Grade 1	Grade 2	Grade 3	Grade 4	Grade 5
Transaminase elevation	20 (32.8)	6 (9.8)	4 (6.6)	10 (16.4)	0	0
Anemia	15 (24.6)	0	10 (16.4)	5 (8.2)	0	0
Asthenia	13 (21.3)	6 (9.8)	5 (8.2)	1 (1.6)	0	1 (1.6)
Fatigue	13 (21.3)	8 (13.1)	3 (4.9)	2 (3.3)	0	0
Hypothyroidism	13 (21.3)	3 (4.9)	9 (14.8)	0	1 (1.6)	0
Back pain	8 (13.1)	2 (3.3)	5 (8.2)	1 (1.6)	0	0
Cough	8 (13.1)	3 (4.9)	5 (8.2)	0	0	0
Decreased appetite	8 (13.1)	6 (9.8)	1 (1.6)	1 (1.6)	0	0
Nausea	8 (13.1)	7 (11.5)	1 (1.6	0	0	0
Abdominal pain	7 (11.5)	2 (3.3)	5 (8.2)	0	0	0
Malignant neoplasm progression	7 (11.5)	0	0	1 (1.6)	0	6 (9.8)

NOTE: Values are number (%) of patients.

Supplementary Table 4. Patient disposition

Variable	*N* = 61
Enrolled and treated	61 (100)
Treatment ongoing	4 (6.6)
Discontinued treatment	57 (93.4)
Documented radiographic PD	41 (67.2)
Clinical progression	8 (13.1)
AE	6 (9.8)[a]
Death	1 (1.6)
Other	1 (1.6)

NOTE: Values are number (%) of patients.

Abbreviations: AE, adverse event; ALT, alanine aminotransferase; AST, aspartate aminotransferase; PD, progressive disease.

[a]Six patients discontinued due to TEAEs, three (4.9%) of whom discontinued due to grade 3 transaminase elevations (elevated ALT and AST and hepatotoxicity in one patient; elevated ALT and AST in one patient; elevated ALT alone in one patient). Other TEAEs leading to discontinuation were immune-mediated nephritis, pneumonitis, and malignant neoplasm progression (each n = 1).

Supplementary Figure 1. Generation of GEN1046 and target binding characteristics. A, Generation of GEN1046 by cFAE of Fc-silenced mAb-PD-L1 and mAb-4-1BB. The parental Abs contain matched mutations in the CH3 domain (F405L/K409R) that drive heterodimerization of the Fab arms and formation of bispecific molecules during cFAE as well as Fc-silencing mutations (L234F, L235E, and D265A) that abrogate binding to FcγR and C1q. **B**, Binding of FcγRIIa, -IIb and -RIIIa dimers to GEN1046 was determined by ELISA. IgG1-PD-L1-F405L (without Fc-silencing mutations; active Fc ctrl) was included as positive control. Data shown are mean ± SD from one representative experiment (*n* = 3). **C**, Binding of the PD-L1-specific Fab arm of GEN1046 (bsAb-PD-L1×ctrl) and control Abs to *in vitro* IFN-γ–stimulated PBMCs was analyzed by flow cytometry. Data shown are MFI ± SD within the $CD3^{-}CD20^{-}HLA\text{-}DR^{+}$ cell gate of triplicate measurements from one representative donor (*n* = 2). **D**, Binding of the 4-1BB-specific Fab arm of GEN1046 (bsAb-ctrlx4-1BB) and control Abs to *in vitro*–activated PBLs was analyzed by flow cytometry. Data shown are MFI ± SD on CD3+4-1BB+ T cells of triplicate measurements from one representative donor (*n* = 3). **E**, Blockade of recombinant 4-1BBL binding to K562_h4-1BB cells by GEN1046 was analyzed by flow cytometry. Ab, antibody; cFAE, controlled Fab arm exchange; MFI, median fluorescent intensity.

A

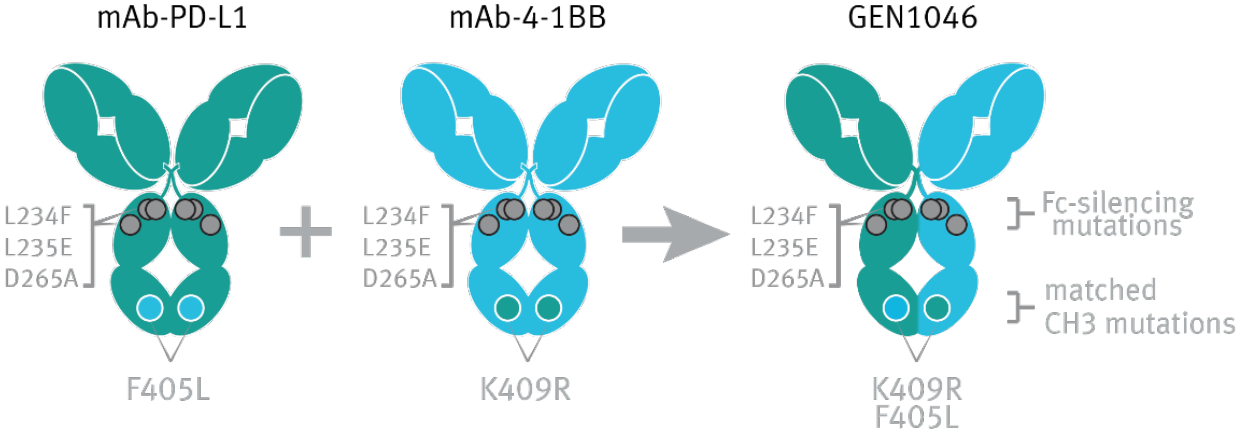

B

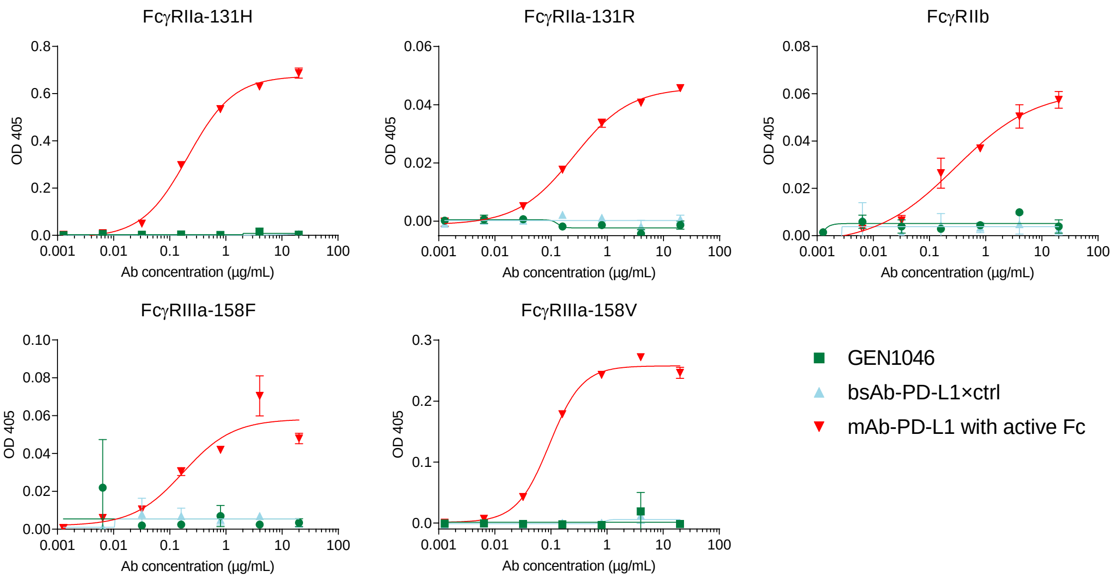
FcγRIIa-131H
FcγRIIa-131R
FcγRIIb
FcγRIIIa-158F
FcγRIIIa-158V
OD 405
Ab concentration (μg/mL)
0.001
0.01
0.1
1
10
100
GEN1046
bsAb-PD-L1×ctrl
mAb-PD-L1 with active Fc

C

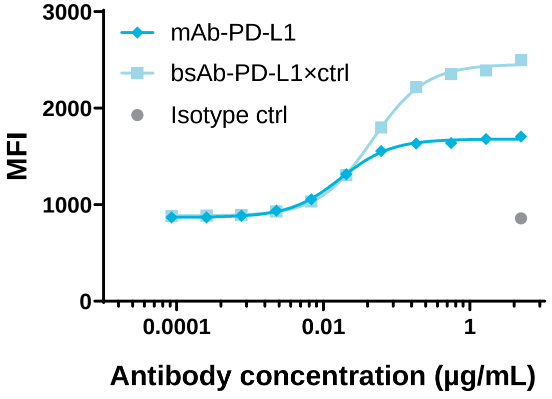
mAb-PD-L1
bsAb-PD-L1×ctrl
Isotype ctrl
MFI
3000
2000
1000
0
0.0001
0.01
1
Antibody concentration (μg/mL)

D

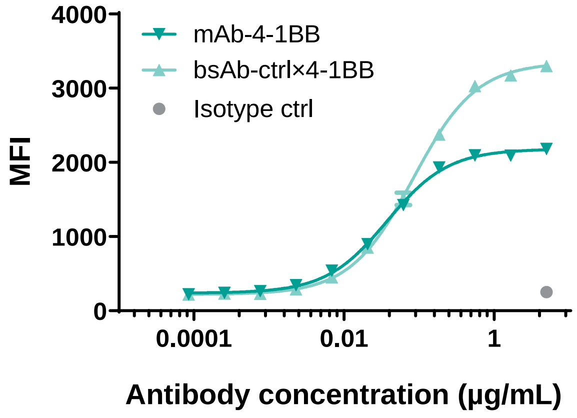
mAb-4-1BB
bsAb-ctrl×4-1BB
Isotype ctrl
MFI
4000
3000
2000
1000
0
0.0001
0.01
1
Antibody concentration (μg/mL)

E

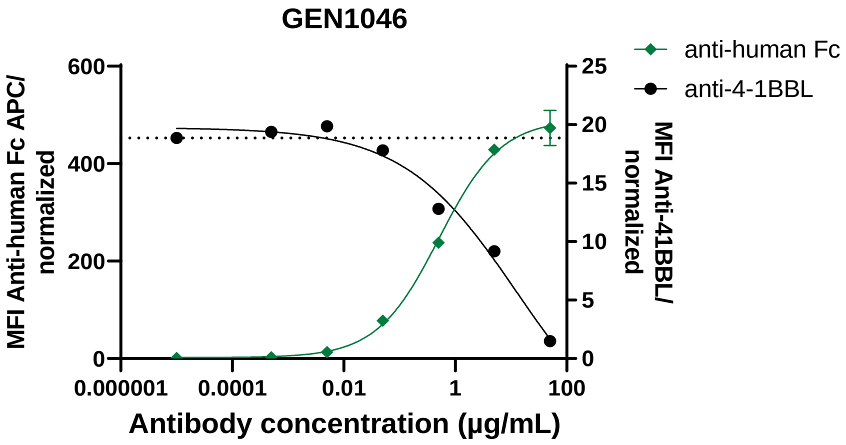
GEN1046
anti-human Fc
anti-4-1BBL
MFI Anti-human Fc APC/ normalized
MFI Anti-41BBL/ normalized
600
400
200
0
25
20
15
10
5
0
0.000001
0.0001
0.01
1
100
Antibody concentration (µg/mL)

Supplementary Figure 2. GEN1046 T-cell effector functions. A, Simultaneous binding of GEN1046 to CellTrace Violet-labeled K562_hPD-L1 cells and CellTrace FarRed-labeled K562_h4-1BB cells was analyzed by flow cytometry. Double-positive doublets were quantified as percentage within the live cell population (mean ± SD; triplicate wells). **B,** Representative images of DC/T-cell cocultures incubated with GEN1046 or the combination of bsAb-PD-L1×ctrl and bsAb-ctrl×4-1BB and stained with anti-human Fc (in magenta) and LFA-1 (in green) antibodies. The arrow indicates the cell for which the staining of GEN1046 was quantified in C. The red line represents the interface between the DC and T cell. Nuclei were counterstained with Hoechst stain (in blue). Scale bars: 10 µm. **C,** Intensity of the staining at the DC–T-cell interface, indicated by the dashed red line, was measured for both LFA1 and GEN1046 or the combination of bsAb-PD-L1xctrl and bsAb-ctrlx4-1BB. ****$P < 0.0001$, Mann–Whitney U test. **D**, GEN1046-enhanced $CD4^+$ T-cell proliferation in vitro. CFSE-labeled human PBMCs were stimulated with anti-CD3 0.1 µg/mL and incubated with GEN1046 or control antibodies (0.2 µg/mL) for 4 days. CFSE dilution in $CD4^+$ T cells was analyzed by flow cytometry and the expansion index was calculated. Data shown are the fold change expansion index of treatment groups relative to untreated cells, of individual donors, and mean ± SD (n = 11). **E-F,** Polyclonal T-cell proliferation assay with GEN1046 treatment (0.2 µg/mL) or no antibody treatment (medium), either in the presence or absence of anti-CD3 (0.1 µg/mL). Data shown are histograms for CFSE dilution in CD8+ and CD4+ T cells of one representative donor (E) and expansion indices of CD8+ and CD4+ T cells from three donors (F), with the shape of symbols indicating data points corresponding to individual donors. **G-H**, Antigen-specific T-cell proliferation assay with GEN1046 treatment (0.2 µg/mL) or no antibody treatment (medium), in co-culture with either CLDN6-electroporated or non-electroporated iDC. Data shown are histograms of CFSE dilution in CD8+ T cells of one representative donor (G) and expansion indices of CD8+ T cells from three donors (H), with the shape of symbols indicating data points corresponding to individual donors. **I**, Cytokine concentrations in supernatant taken after 48 h

from cultures. CD8^{+} T cells were electroporated with RNA encoding an HLA-A2/CLDN6-specific TCR and PD-1 (5 µg), labeled with CFSE, and cocultured with autologous DCs electroporated with CLDN6-encoding RNA in presence of the GEN1046 or control antibodies (0.2 µg/mL) for 4 days.

A

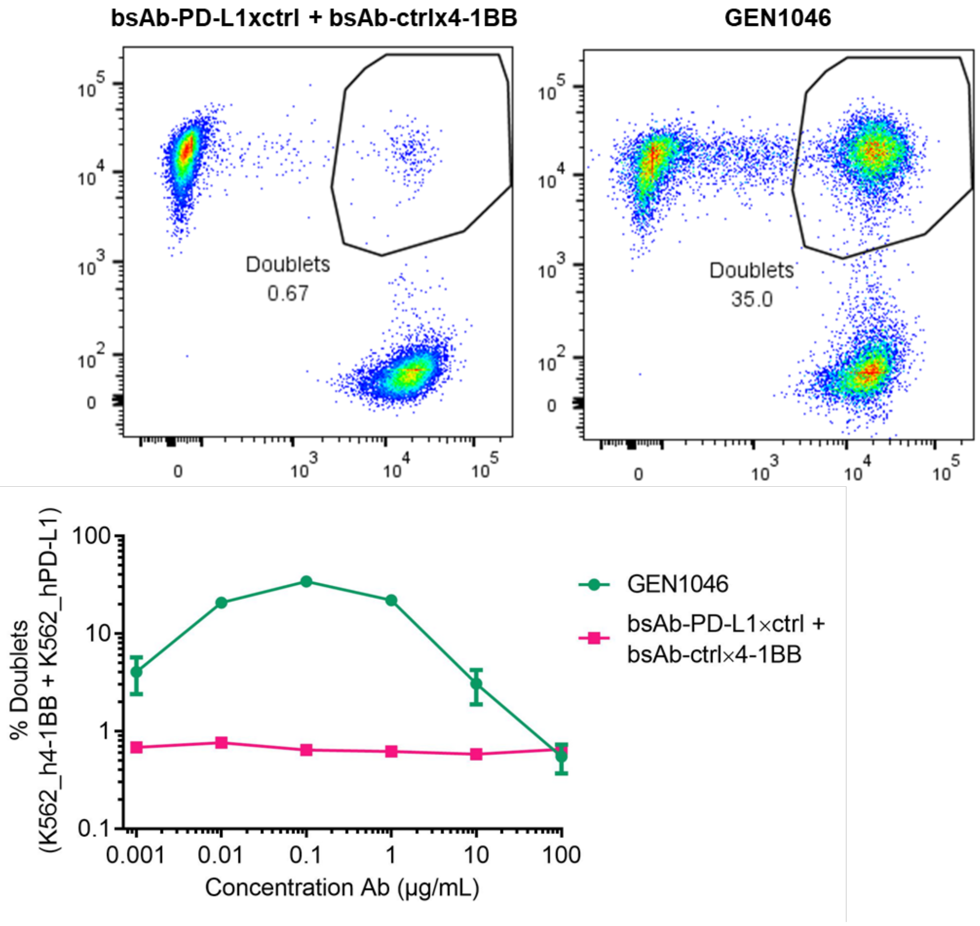

B

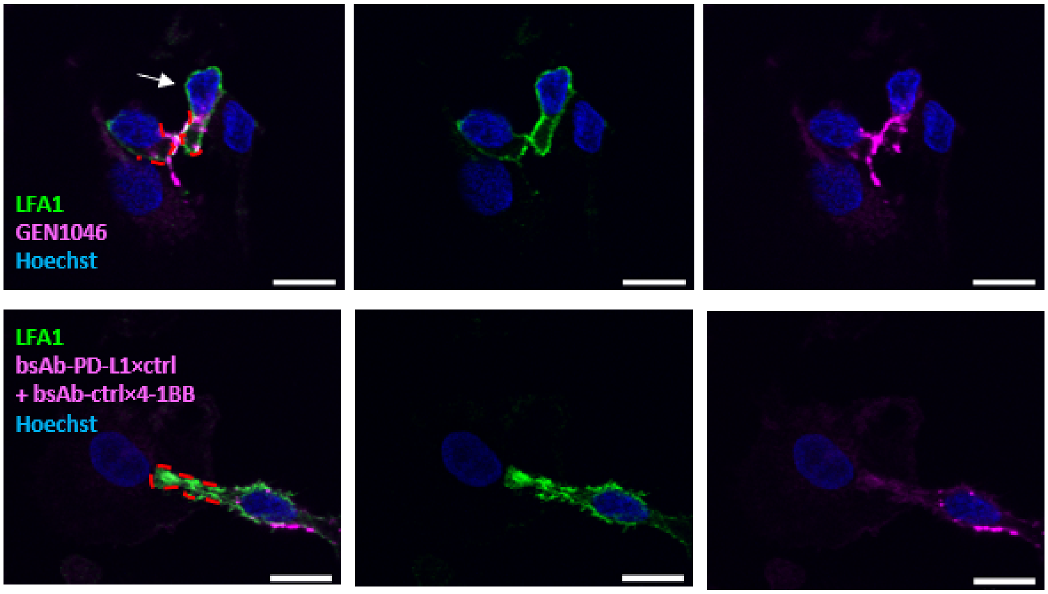
LFA1
GEN1046
Hoechst
LFA1
bsAb-PD-L1×ctrl
+ bsAb-ctrl×4-1BB
Hoechst

C

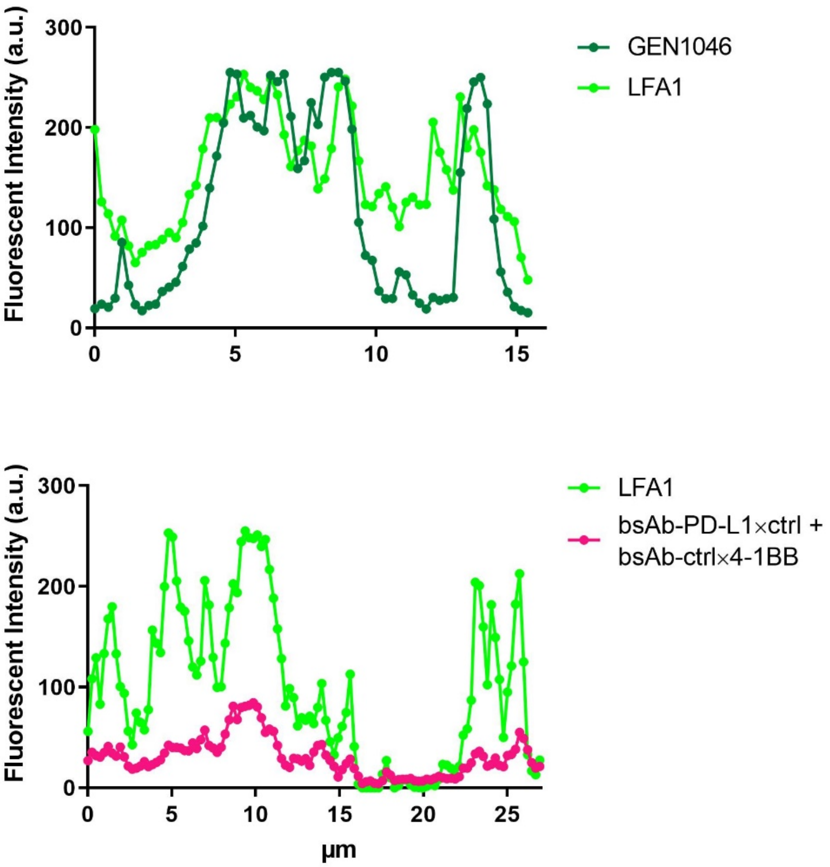
Fluorescent Intensity (a.u.)
300
200
100
0
0
5
10
15
GEN1046
LFA1
Fluorescent Intensity (a.u.)
300
200
100
0
0
5
10
15
20
25
µm
LFA1
bsAb-PD-L1×ctrl +
bsAb-ctrl×4-1BB

D

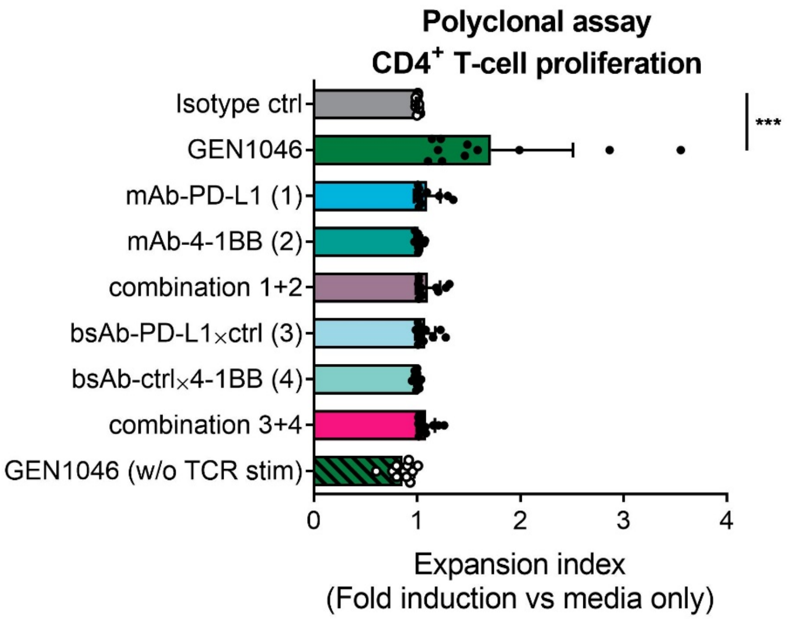
Polyclonal assay
CD4+ T-cell proliferation
Isotype ctrl
GEN1046
mAb-PD-L1 (1)
mAb-4-1BB (2)
combination 1+2
bsAb-PD-L1×ctrl (3)
bsAb-ctrl×4-1BB (4)
combination 3+4
GEN1046 (w/o TCR stim)
***
0
1
2
3
4
Expansion index
(Fold induction vs media only)

E

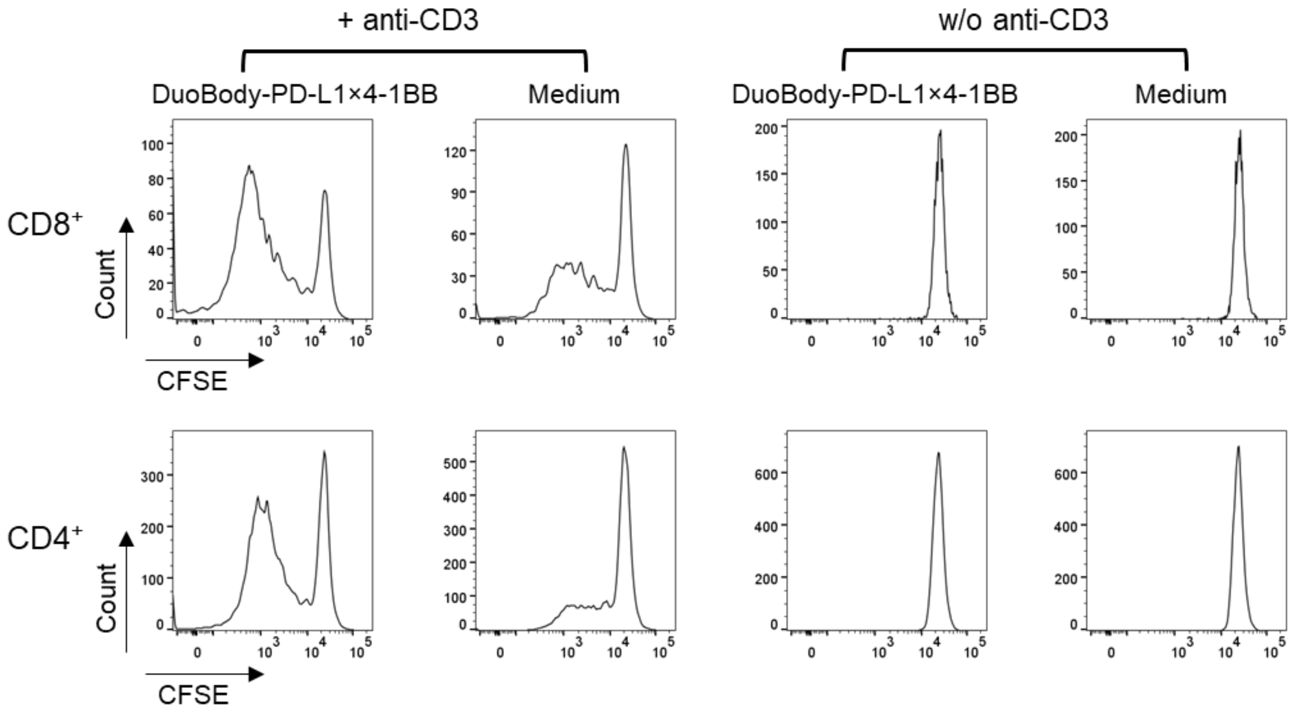
+ anti-CD3
w/o anti-CD3
DuoBody-PD-L1×4-1BB
Medium
DuoBody-PD-L1×4-1BB
Medium
CD8+
CD4+
Count
CFSE

F

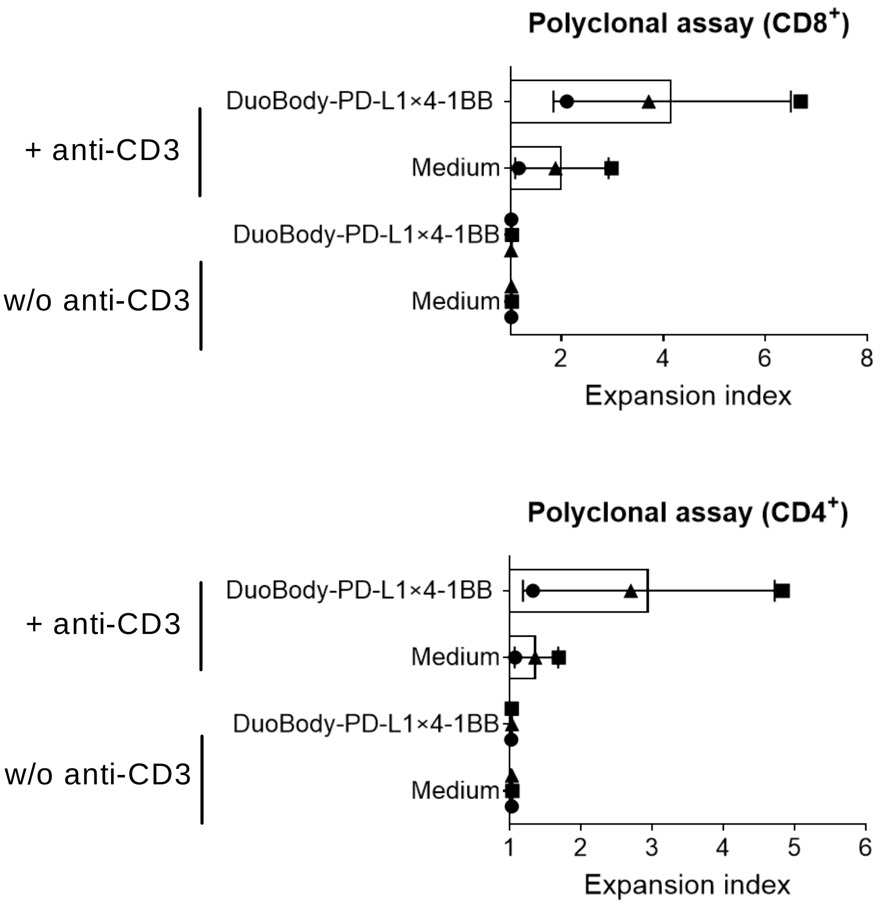
Polyclonal assay (CD8$^+$)
+ anti-CD3
w/o anti-CD3
DuoBody-PD-L1×4-1BB
Medium
DuoBody-PD-L1×4-1BB
Medium
2 4 6 8
Expansion index
Polyclonal assay (CD4$^+$)
+ anti-CD3
w/o anti-CD3
DuoBody-PD-L1×4-1BB
Medium
DuoBody-PD-L1×4-1BB
Medium
1 2 3 4 5 6
Expansion index

G

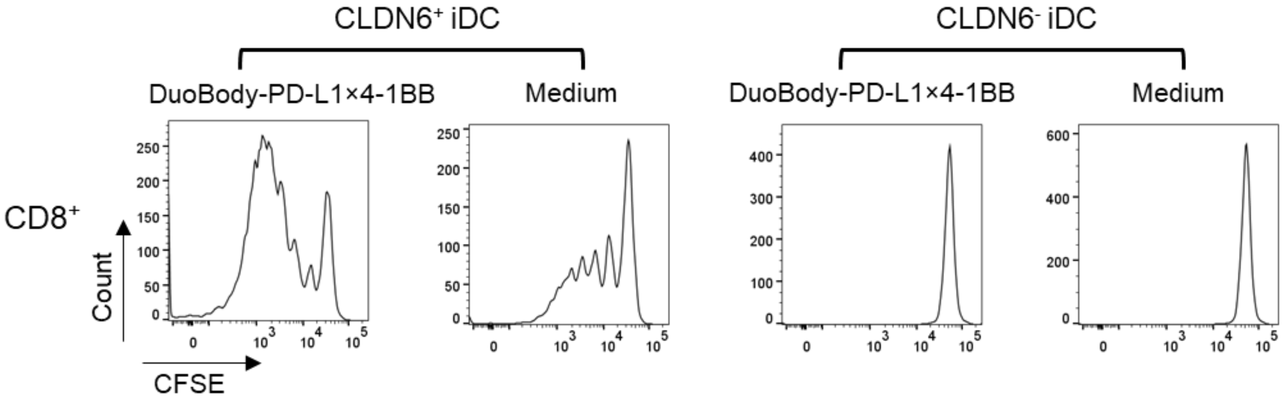

CLDN6+ iDC
CLDN6- iDC
DuoBody-PD-L1×4-1BB
Medium
DuoBody-PD-L1×4-1BB
Medium
CD8+
Count
CFSE

H

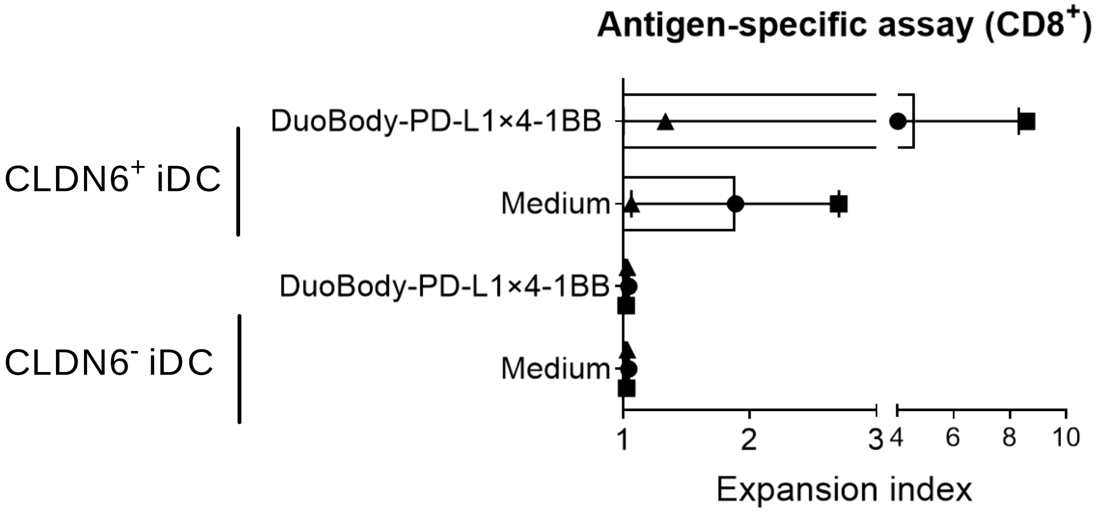

Antigen-specific assay (CD8+)
CLDN6+ iDC
CLDN6- iDC
DuoBody-PD-L1×4-1BB
Medium
DuoBody-PD-L1×4-1BB
Medium
1
2
3
4
6
8
10
Expansion index

I

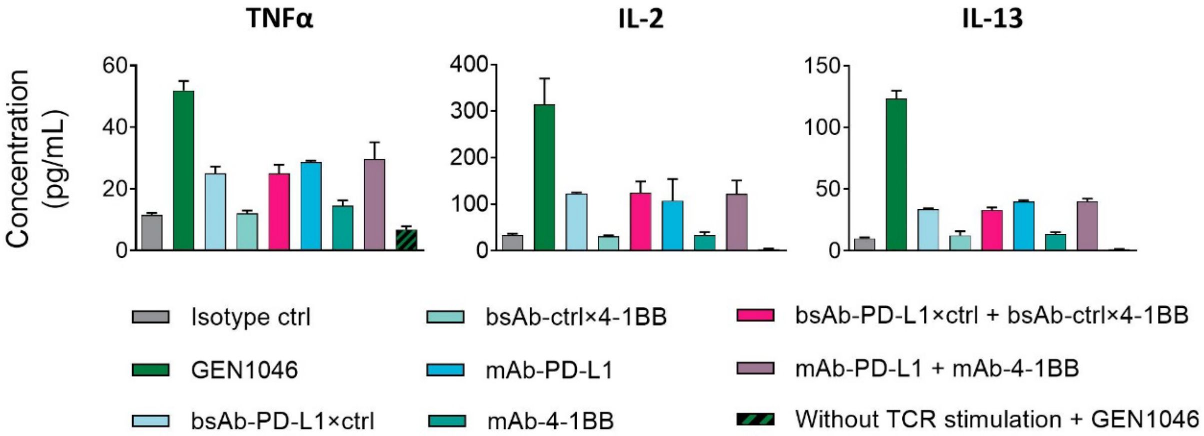
TNFα
IL-2
IL-13
Concentration (pg/mL)
60
40
20
0
400
300
200
100
0
150
100
50
0
Isotype ctrl
GEN1046
bsAb-PD-L1×ctrl
bsAb-ctrl×4-1BB
mAb-PD-L1
mAb-4-1BB
bsAb-PD-L1×ctrl + bsAb-ctrl×4-1BB
mAb-PD-L1 + mAb-4-1BB
Without TCR stimulation + GEN1046

Supplementary Figure 3. Change in sum of diameters of target lesions from baseline over time in all patients (*n* = 61). NA, not applicable; NE, not evaluable; uCR, unconfirmed CR.

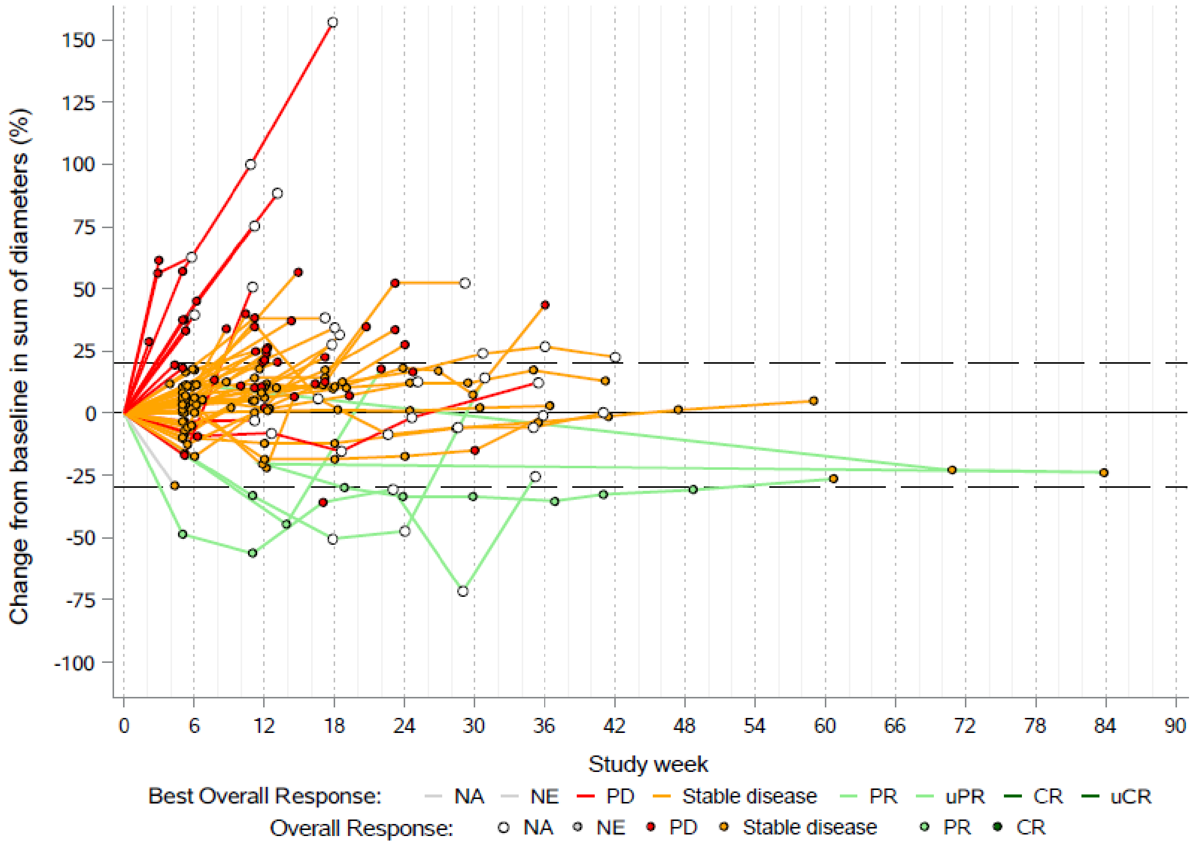

Supplementary Figure 4. Immunohistochemical staining of archival tumor tissue in the presented case studies. A, Patient with NSCLC whose best response to last therapy (bevacizumab plus an investigational drug) was stable disease. Biopsy was obtained approximately 4 months before first dose of GEN1046. **B**, Patient with ovarian cancer whose best response to last therapy (bevacizumab) was stable disease. Biopsy was obtained approximately 22 months before first dose of GEN1046.

A

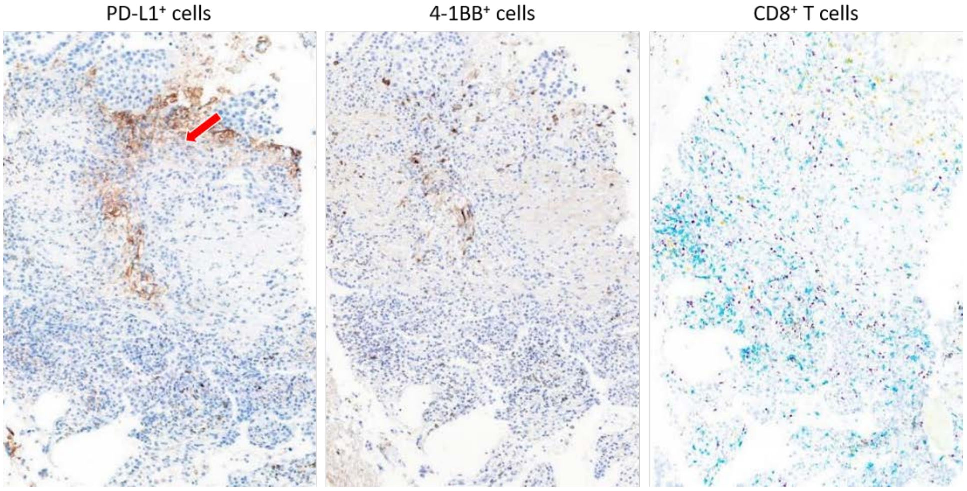

B

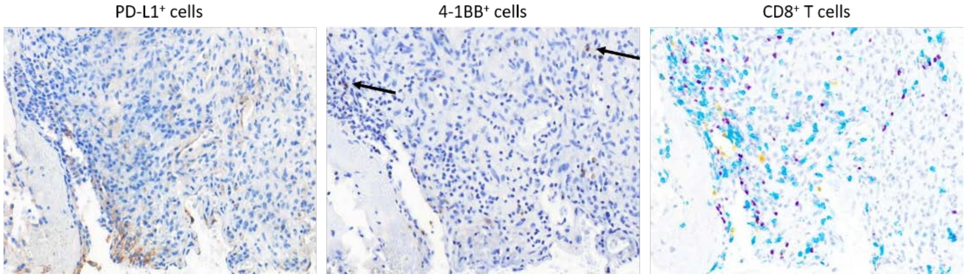

SUPPLEMENTARY METHODS

Antibodies, reagents, and cell lines

Commercial antibodies are listed in Supplementary Methods Table 1. In general, research antibodies were produced at Genmab in a human IgG1 backbone and included Fc-silencing mutations (L234F, L235E, D265A) and matched DuoBody mutations in the CH3 region for controlled Fab arm exchange (F405L and K409R), unless otherwise specified (Supplementary Methods Table 2). Clinical-grade atezolizumab, pembrolizumab, and nivolumab were used in experiments unless specified as an analog. In some experiments, an analog of atezolizumab was used that comprised identical complementarity-determining region 3 sequences as clinical atezolizumab in a human IgG1 backbone with Fc-silencing and DuoBody mutations as above.

Cell culture and media buffers are listed in Supplementary Methods Table 3.

Generation of parental antibodies mAb-PD-L1 and mAb-4-1BB

The PD-L1-specific antibody mAb-PD-L1 was generated by hybridoma technology using the transgenic OmniRat (Ligand Pharmaceuticals, Emeryville, CA) antibody platform. To obtain a fully human antibody, the selected V_L and V_H sequences were subsequently cloned in a human backbone that also contained human IgG1 constant heavy chain regions in which the DuoBody mutation (F405L) and the Fc-silencing L234F, L235E, and D265A (FEA) mutations had been introduced. The 4-1BB-specific antibody mAb-4-1BB was generated by immunization of rabbits and single B-cell cloning. To obtain a humanized antibody, the selected V_L and V_H sequences were humanized using germline humanization (complementarity-determining region–grafting) technology and subsequently expressed in a human backbone that also contained human IgG1 constant heavy chain regions in which the DuoBody mutation (K409R) and the Fc-silencing FEA mutations had been introduced. Both mAb-PD-L1 and mAb-4-1BB were selected from a panel of PD-L1- and 4-1BB-specific antibodies based on comprehensive data, including target binding characteristics as well as biologic activity in a bsAb format.

Fcγ receptor binding assays

ELISA plates (Greiner Bio-One [Kremsmünster, Austria], 655092) were coated with 1-µg/mL goat F(ab')2-anti-human (Jackson ImmunoResearch [West Grove, PA], 109-006-097; RRID, AB_2337550), blocked with 0.2% bovine serum albumin Fraction V (Roche [Basel, Switzerland], 10735086001), incubated with antibodies at room temperature for 1 h, followed by 1-µg/mL HIS-

and biotin acceptor peptide (BAP)–tagged recombinant dimeric Fcγ receptor constructs (diFcɣR HisBAP; previously described (1) and produced at Genmab) at room temperature for 1 h, streptavidin-poly-horseradish peroxidase (HRP; CLB, M2032) at room temperature for 30 minutes, and 2,2'-azino-bis(3-ethylbenzothiazoline-6-sulphonic acid; Roche, 11112422001) at room temperature for 10–30 minutes. Absorption at OD405 was measured on an ELx808 Absorbance Microplate Reader (BioTek Instruments, Winooski, VT).

Biolayer interferometry

Target-binding affinity of PD-L1–specific and 4-1BB–specific antibodies was determined by biolayer interferometry on an Octet HTX instrument (FortéBio, Fremont, CA). Experiments were carried out while shaking at 1000 rpm at 30°C.

For binding of PD-L1–specific antibodies, anti-human IgG Fc Capture biosensors (FortéBio, 18-5060) were loaded with mAb-PD-L1 (1 µg/mL; Supplementary Methods Table 2). After a baseline measurement (300 s) in Sample Diluent (FortéBio, 18-1104 and 18-1048), association (1000 s) and dissociation (1000 s) of recombinant soluble human HisC-tagged PD-L1 ECD (PDLoneECDHisCtag, produced at Genmab) or recombinant cynomolgus monkey PD-L1-His protein (Acro Biosystems [Newark, NJ], PD1-C52H4) was determined. For binding of 4-1BB–specific antibodies, activated amine-reactive second-generation biosensors (FortéBio, 18-5092) were loaded with recombinant human 4-1BB ECD fused to a His-tag (4-1BB-His, 2.5 µg/mL; Sino Biological [Beijing, China], 10041-H08H) or recombinant cynomolgus monkey 4-1BB-His (Sino Biological, 90847-K08H). After a baseline measurement (1000 s) in Sample Diluent, association (200 s) and dissociation (2000 s) of bsAb-ctrl×4-1BB was determined.

Data were acquired using Data Acquisition Software (FortéBio, v9.0.0.49d) and analyzed with Data Analysis Software (FortéBio, v9.0.0.14). Data were fitted with the 1:1 Global Full fit model.

Cell-based binding and blocking assays

Binding of PD-L1–specific antibodies to primary $CD3^-CD20^-HLA\text{-}DR^+$ cells was analyzed by flow cytometry using PBMCs that had been stimulated overnight with 2000 U/mL recombinant human IFN-γ (PeproTech [Cranbury, NJ], AF-300-02). Binding of 4-1BB–specific antibodies to primary $CD3^+$ T cells was analyzed by flow cytometry using the $CD14^-$ fraction of PBMCs that had been stimulated with Dynabeads Human T-Activator CD3/CD28 (Life Technologies, 11132D) for 48 h. For assessment of receptor/ligand blocking, K562_h4-1BB cells were incubated with GEN1046, followed by washing and incubation with recombinant 4-1BBL (Acro

Biosystems, 41L-H5265). Subsequently, cell-bound GEN1046 and 4-1BBL were stained with anti-human IgG (Jackson ImmunoResearch, 109-136-170; RRID, AB_2337695) and noncompeting anti–4-1BBL (BioLegend, 311504; RRID, AB_314883) and analyzed by flow cytometry. Simultaneous binding of GEN1046 to cells expressing PD-L1 or 4-1BB was analyzed by flow cytometry assessment of doublet formation after coincubation of K562-hPD-L1 cells labeled with CellTrace Violet (Thermo Fisher, C34557) and K562-h4-1BB cells labeled with CellTrace FarRed (Thermo Fisher, C34564), for 15 minutes at 37 °C.

Immunofluorescence and live cell imaging

For immunofluorescence, cocultured DCs with CD8$^+$ T cells were fixed for 10 minutes at room temperature in 4% paraformaldehyde, permeabilized with 0.25% Triton-X/PBS, blocked in 2% bovine serum albumin/PBS, and stained for cell surface antigens using commercial antibodies and with Hoechst (Life Technologies, H3570). Coverslips were washed and mounted with Immomount media (Life Technologies, 9990402). Images were acquired at the confocal microscope Leica Sp8 (Leica, Wetzlar, German).

For live cell imaging, time-lapse videos were performed at 37 °C and 5% CO_2 at the Nikon Eclipse TiE microscope (Nikon, Tokyo, Japan) with a time frame of 15 minutes. To facilitate the visualization of contact events and the formation of synapses between CD8$^+$ T cells and DCs, a sequence of still images was recorded using the semiautomatized macro. Time 0 was defined as time of first contact between DC and CD8$^+$ T cells.

Image analysis was performed using the Fiji (ImageJ 1.52p) software. The intensity profile of the fluorescence at the synapse was generated selecting the single channels *Image* > *Color* > *Channel Tool*. At the interface between the DC and the CD8$^+$ T cell a line was generated selecting the *segmented line* tool. By selecting *Analyze* > *Plot profile* an intensity profile was generated. For the live imaging, cells were manually followed, and the number of contacts and contact duration was manually determined during the videos.

RNA sequencing

For TCR repertoire sequencing, total RNA was isolated from tumor tissue or snap-frozen cultured TILs using the RNeasy Mini kit (Qiagen [Hilden, Germany], 74104). Libraries were generated with the SMARTer human TCR-αβ profiling kit (Takara Bio [Shiga, Japan], 635015) and were sequenced using the MiSeq system (Illumina, San Diego, CA). Data were analyzed using MiXCR (MiLaboratory, Sunnyvale, CA) (2) and VDJtools (3).

Flow cytometry

Cultured cells or mechanically dissociated splenocytes were stained for cell surface antigens using commercial antibodies for 15–30 minutes at 4 °C. For intracellular staining, cells were fixed and permeabilized followed by intracellular staining for 30 minutes at 4 °C. Viable cells were distinguished by staining with 7-aminoactinomycin D (Beckman Coulter [Brea, CA], A07704), eFluor780 (eBioscience, 65-0865-18), or LIVE/DEAD Fixable Near-IR Dead cell stain kit (Thermo Scientific, L10119). Data were acquired on a LSRFortessa X-20, FACSCanto, or FACSCelesta flow cytometer (BD Biosciences). For cell counting, CountBright counting beads (Thermo Fisher, C36995) were added to the cells prior to acquisition. Data were analyzed using FlowJo software v10.3 (BD Biosciences), BD FACSuite software v1.0.5 (BD Biosciences), or Kaluza Analysis software (Beckman Coulter). Proliferation analysis based on CFSE dilution was performed using the proliferation modeling tool from FlowJo, the generation peaks were automatically fitted, and expansion index values were calculated.

Cytokine analysis

Cell culture supernatants were analyzed using the V-PLEX Proinflammatory Panel 1 Human Kit (Meso Scale Diagnostics [Rockville, MD], K15049D-2) for evaluation of human cytokines and using the V-PLEX Proinflammatory Panel 1 mouse Kit (Meso Scale Diagnostics, K15048D-2) and the V-PLEX Cytokine Panel 1 mouse Kit (Meso Scale Diagnostics, K15245D-2) for evaluation of mouse cytokines on a MESO QuickPlex SQ 120 instrument (Meso Scale Diagnostics, R31QQ-3), according to manufacturer instructions.

Immunohistochemistry

Tumors were dissected, fixed in formalin, and paraffin embedded and sectioned (4 µm). For histologic assessment, tumor sections were deparaffinized and stained with the Tissue-Tek Prisma H&E Stain Kit (Sakura [Torrance, CA], 6190) using the Tissue-Tek Prisma Plus Automated Slide Stainer (Sakura). For evaluation of $CD3^+$, $CD8^+$ T cells, or $FoxP3^+$ cells within the tumor, sections were deparaffinized and antigens were retrieved using CC1 buffer (Roche, 950-124), followed by quenching of endogenous peroxidase (Dako Agilent, S2003) and blocking of aspecific binding sites with blocking buffer (Roche, 05268869001) using the Roche Ventana Discovery (DISC) autostainer platform. Sections were incubated with primary antibodies

(Supplementary Methods Table 1), which were detected using antirabbit immunohistochemistry detection kits: for CD3 with only antirabbit DISC, Omnimap (Roche, 05269679001) for CD8 and FoxP3 sequentially with DISC anti-rabbit HQ (Roche, 07017812001) and DISC, and amplification for anti-HQ HRP Multimer (Roche, 06442544001). HRP was visualized using 3,3'-diaminobenzidine (ChromoMap DAB; Roche, 05266645001) according to manufacturer instructions. Nuclei were counterstained by incubation with Mayer hematoxylin. Staining specificity was controlled by incorporating isotype control staining on consecutive tissue sections. Stained slides were subjected to whole slide imaging (FluoFloor), and whole slide images were uploaded to and analyzed with Halo software (Indica Labs, Albuquerque, NM) using preprogrammed software analysis tools to determine $CD3^+$, $CD8^+$ and $FoxP3^+$ cells (CytoNuclear v2.0.9). $CD3^+$, $CD8^+$, and $FoxP3^+$ cells were subsequently expressed as number of cells/mm^2.

Toxicity study in cynomolgus monkeys

A good laboratory practice toxicity study evaluated the tolerability and potential toxicity of GEN1046 in Mauritian cynomolgus macaques at Covance Preclinical Services. Study procedures complied with the German Animal Welfare Act and were approved by the local Institutional Animal Care and Use Committee.

GEN1046 at 0, 1, 5 or 30 mg/kg was administered on days 1 and 22 (5 animals/sex/group) via intravenous infusion. Toxicity assessments included clinical and neurobehavioral observations, body weight, ophthalmology, electrocardiogram, blood pressure and respiratory rate, clinical and anatomical pathology, cytokines, and immunophenotyping. Necropsies were conducted on three animals per sex per group on day 29 (terminal) or two animals per sex per group on day 50 (recovery).

Analysis of serum transaminase and cytokine levels in peripheral blood and immunophenotyping of peripheral blood, lymph nodes and spleen of cynomolgus monkeys

Blood samples (1.5 mL) for clinical chemistry parameters were collected from fasted animals once during the predose phase, on days 1 and 22 before dosing (0 h), on days 8 and 29 (day of necropsy), and on all surviving animals during the last week of the recovery phase. AST and ALT levels were determined in serum from whole blood.

Statistical analysis of preclinical data

Wilcoxon sum rank or Mann–Whitney U tests were used for pairwise comparisons. Friedman test or one-way ANOVA with Dunnett multiple comparisons test were used as indicated in the figure legends.

Clinical study

Selection of starting dose

The human dose equivalent to the NOAEL dose with a comparable area under the concentration-time curve was predicted using a pharmacokinetic model from cynomolgus monkey data with an adequate safety factor applied, resulting in an estimated dose of approximately 0.3 mg/kg. A flat starting dose of 25 mg Q3W corresponding to 0.3 mg/kg was selected, assuming a median human body weight of approximately 80 kg.

Dose reductions and interruptions

Per protocol, treatment with GEN1046 was interrupted in patients who experienced grade 3 transaminase elevations; if the event resolved to grade 1 or to baseline levels within 14 days, GEN1046 could have been continued. Patients who experienced grade 4 transaminase elevations were required to permanently discontinue GEN1046.

Flow cytometry

Blood samples were collected just prior to administration of GEN1046 in cycle 1 and cycle 2 and at the following timepoints after administration in each cycle: day 2, day 3, day 8 and day 15. Cellular subsets in the blood were evaluated using antibody panels described earlier and a Becton Dickinson FACSCanto™ flow cytometer equipped with FacsDiva software version 8.0.1.

Cytokines/chemokines analysis

Blood samples were collected just prior to administration of GEN1046 and at 2 h, 4-6 h, day 2, day 3, and day 8 after administration in cycles 1 and 2. Cytokines and chemokines were

evaluated using V-PLEX Plus Human Biomarker 40-Plex kit (Meso Scale Diagnostics LLC, K15209G-1) on a Meso Sector S600 instrument (Meso Scale Diagnostics, LLC., IC0AA-0).

Immunohistochemistry of tumor samples

Expression of markers of interest was assessed by immunohistochemistry in archival tumor specimens. PD-L1 expression was assessed by 22C3 pharmDx assay (HistoGeneX, Belgium); 4-1BB (CD137 or TNFRSF9) expression was assessed by clone D2Z4Y on Ventana Benchmark Ultra, and detection of CD8 and Granzyme B were assessed by a multiplex chromogenic assay on Ventana Discovery Ultra.

Supplementary Methods Tables

Supplementary Methods Table 1. Commercial antibodies

Target	Label	Clone	Supplier	Catalog no.	RRID
CLDN6	DyLight650	IMAB 027	Ganymed	NA	NA
TCRβ	BV421	H57-597	BD Biosciences	562839	AB_2737830
PD-1	APC	eBioJ105	Thermo Fisher Scientific	17-2799-42	AB_11063701
IgG2,λ	BV421	Ha4/8	BD Biosciences	562629	AB_2869429
IgG1,κ	APC	MOPC-21	BD Biosciences	555751	AB_398613
CD3	BV421	SP34-2	BD Biosciences	562877	AB_2737860
CD20	APC	2H7	BD Biosciences	559776	AB_398670
HLA-DR	PerCP-Cy5.5	G46-6	BD Biosciences	560652	AB_1727529
IgG, Fcγ Fragment Specific	AF488	—	Jackson ImmunoResearch	109-546-098	AB_2337850
CD137 (4-1BB)	APC	4B4-1[2]	BD Biosciences	550890	AB_398477
LFA-1	UC	HI111	Thermo Fisher Scientific	14-0119-82	AB_467122
AffiniPure F(ab') Fragment Goat Anti-Mouse IgG (H+L)	AF488	polyclonal	Jackson ImmunoResearch	115-546-146	AB_2338868
Anti-human Fc	FITC/PE	ns	Beckman Coulter	A07794	NA
CD3	—	UCHT1	R&D systems	MAB100-500	AB_357359
CD4	PE	SK3	BioLegend	344606	AB_1937246
CD8	PE-Cy7	RPA-T8	TONBO Biosciences	60-0088	AB_2621834
CD8	PE	RPA-T8	TONBO Biosciences	50-0088	AB_2621744
GrzB	PE	GB11	BD Biosciences	561142	AB_10561690
CD107a	AF647	H4A3	BioLegend	328611	AB_1227507
CD3	PE-Cy7	UCHT1	eBioscience	25-0038	NA
CD4	FITC	M-T466	Miltenyi Biotec	130-080-501	AB_244326
CD56	APC	CMSSB	Thermo Fisher Scientific	17-0567-042	AB_10597454
HLA A,B,C	N/A	W6/32	BioLegend	311402	AB_314871
CD3	UC	2GV6	Ventana	790-4341	AB_2335978
CD8α	UC	D4W2Z	Cell Signaling Technology	98941	AB_2756376
FoxP3	UC	D2W8E	Cell Signaling Technology	98377	AB_2747370

IgG XP®	UC	DA1E	Cell Signaling Technology	3900S	AB_1550038
CD45	BV785	30-F11	BioLegend	103149	AB_2564590
CD3	BUV395	17A2	BD Biosciences	740268	AB_2687927
CD4	BV421	GK1.5	BioLegend	100438	AB_11203718
CD8	PE-eFluor610	53-6.7	Thermo Fisher Scientific	61-0081-82	AB_2574524
Foxp3	PE	FJK-16s	Thermo Fisher Scientific	12-5773-82	AB_465936
CD19	BV711	6D5	BioLegend	115555	AB_2565970
CD45	PerCP/Cy 5.5	30-F11	BioLegend	103132	AB_893340
CD8a	PE	53-6.7	BioLegend	100708	AB_312747
H-2D^{b} Adpgk Neoepitope tetramer	APC	NA	MBL	TB-5113-2	NA
PD-1	BV711	29F.1A12	BioLegend	135231	AB_2566158
CD44	FITC	IM7	BioLegend	103022	AB_493685
CD62L	BV785	MEL-14	BioLegend	104440	AB_2629685

Abbreviations: AF, AlexaFluor; APC, allophycocyanin; BV, brilliant violet; Cy: cyanine; FITC, fluorescein isothiocyanate; PE, phycoerythin; PerCP, peridinin-chlorophyll-protein; RRID, research resource identifier; UC, unconjugated.

Supplementary Methods Table 2. Research antibodies

Antibody	Description	Supplier (batch/catalog no.)
GEN1046	BsAb that contains a PD-L1-specific Fab-arm, a 4-1BB-specific Fab arm and a human IgG1 backbone with Fc-silencing and the DuoBody mutations	Genmab (6371-06-EP)
		Genmab (6371-15-13)
		Genmab (171213_PSM_0014#001)
		Genmab (F19250)
GEN1046 surrogate	BsAb that contains a PD-L1-specific Fab-arm of GEN1046, a non-humanized variant of the 4-1BB-specific Fab arm of GEN1046 and a human IgG1 backbone with Fc-silencing and the DuoBody mutations	Genmab (Plate AD00042781, well C10)
mAb-PD-L1	Bivalent PD-L1-specific mAb in a human IgG1 backbone with Fc-silencing and a DuoBody mutation; parental antibody for GEN1046	Genmab (170524_PSM_0002#001)
		Genmab (2072980-EP)
		Genmab (SG00644/H1)
		Genmab (3967-122-EP)
mAb-PD-L1 (active Fc)	Bivalent PD-L1-specific mAb in an Fc-active human IgG1 backbone	Genmab (171011_PSM_0004#001)
mAb-4-1BB	Bivalent 4-1BB-specific mAb in a human IgG1 backbone with Fc-silencing and a DuoBody mutation; parental antibody for GEN1046	Genmab (4121-131-EP)
		Genmab (4053-090-EP)
mAb-4-1BB (non-humanized)	Bivalent, non-humanized 4-1BB-specific mAb in a human IgG1 backbone with Fc-silencing and a DuoBody mutation	Genmab (3487-106-EP)
bsAb-PD-L1×ctrl	BsAb that contains a PD-L1-specific Fab arm, a non-binding control (ctrl) Fab arm based on IgG-b12, and a human IgG1 backbone with Fc-silencing and the DuoBody mutations	Genmab (170515_PSM_0020#002)
		Genmab (180123_PSM_0029#001)
bsAb-PD-L1×ctrl (A488-labeled)	BsAb that contains a PD-L1-specific Fab arm, a non-binding control (ctrl) Fab arm based on IgG-b12, and a human IgG1 backbone with Fc-silencing and the DuoBody mutations that was labeled with A488	Genmab (171128_PSM_0034#001)
Non-blocking PD-L1 control antibody (A488-labeled)	Bivalent PD-L1-specific mAb that does not cross-block with mAb-PD-L1 in a human IgG1 backbone with Fc-silencing and the DuoBody mutations that was labeled with A488	Genmab (180123_PSM_0033#001)

bsAb-ctrl×4-1BB	BsAb that contains a 4-1BB-specific Fab arm, a non-binding control (ctrl) Fab arm based on IgG-b12, and a human IgG1 backbone with Fc-silencing and the DuoBody mutations	Genmab (170626_PSM_0016#001)
		Genmab (Bis-1035-023)
		Genmab (3593-072-EP)
bsAb-ctrl×4-1BB (non-humanized, A488-labeled)	BsAb that contains a non-humanized variant of the 4-1BB-specific Fab arm of GEN1046, a non-binding control (ctrl) Fab arm based on IgG-b12, and a human IgG1 backbone with Fc-silencing and the DuoBody mutations that was labeled with A488	Genmab (170512_PSM_0024#004)
Non-blocking 4-1BB control antibody (A488-labeled)	Bivalent 4-1BB-specific mAb that does not cross-block with mAb-4-1BB in a human IgG1 backbone with Fc-silencing and the DuoBody mutations that was labeled with A488	Genmab (171106_PSM_0049#001)
Isotype ctrl	Bivalent nonbinding ctrl mAb based on IgG-b12 in a human IgG1 backbone with Fc-silencing and a DuoBody mutation	Genmab (3666-078-EP)
		Genmab (3392-107-EP)
		Genmab (3572-122-EP)
		Genmab (170927_PSM_0023#001)
		Genmab (180704_PSM_0038#001)
		Genmab (181005_PSM_0026#004)
		Genmab (Sf8888-b12-008)
Atezolizumab analog	Bivalent PD-L1-specific antibody based on MPDL3280A in human IgG1 backbone with Fc-silencing and the DuoBody mutations	Genmab (3359-119-EP)
Durvalumab analog	PD-L1 antibody	CrownBio (AB190036)

Supplementary Methods Table 3. Cell culture media and buffers

Name	Composition
X-Vivo^{15} medium	X-VIVO^{15} chemically defined, serum-free hematopoietic cell medium (Lonza, BE02-060Q)
OptiPro SFM medium	OptiPro SFM medium (Thermo Fisher Scientific Europe B.V., 12309050) supplemented with 50 Units penicillin/50 Units streptomycin (Lonza, DE17-603E)
DC medium	RPMI 1640 medium supplemented with 5% plasma-derived human serum [PHS, One Lambda Inc., A25761], 1x minimum essential medium non-essential amino acids [MEM NEAA, Life Technologies GmbH, 11140-035], 1 mM sodium pyruvate [Life Technologies GmbH, 11360-039], 100 ng/mL [Miltenyi Biotec GmbH, 130-093-868] and 50 ng/mL IL-4 [Miltenyi Biotec GmbH, 130-093-924]
T-cell assay medium	Iscove's Modified Dulbecco's Medium [IMDM, Life Technologies GmbH, 12440-053] supplemented with 5% PHS
Transport medium	HypoThermosol® FRS [BioLifeSolutions, 101104] supplemented with 3% Fungizone [Thermo Fisher, 15290-026] and 300 U/mL penicillin, 300 µg/mL streptomycin [pen/strep, Thermo Fisher, 15140-122]
Wash medium	X-Vivo15 medium [Lonza, BE02-060Q] supplemented with 100 U/mL penicillin and 100 µg/mL streptomycin
TIL medium	X-Vivo15 medium supplemented with 10% human serum albumin [HSA, CSL Behring, PZN-00504775], 100 U/mL pen, 100 µg/mL strep, 1% Fungizone and 10 U/mL IL2 [Proleukin®S, Novartis Pharma, PZN-02238131]
FACS buffer	DPBS supplemented with 2% FBS and 2 mM EDTA
DMEM	Hyclone, SH30243.01
Inactivated FBS	ExCell Biology, FSP500

REFERENCES

1. Wines BD, Vanderven HA, Esparon SE, Kristensen AB, Kent SJ, Hogarth PM. Dimeric FcγR ectodomains as probes of the Fc receptor function of anti-influenza virus IgG. J Immunol 2016;197:1507–16.

2. Bolotin DA, Poslavsky S, Mitrophanov I, Shugay M, Mamedov IZ, Putintseva EV, et al. MiXCR: software for comprehensive adaptive immunity profiling. Nat Methods 2015;12:380–1.

3. Shugay M, Bagaev DV, Turchaninova MA, Bolotin DA, Britanova OV, Putintseva EV, et al. VDJtools: unifying post-analysis of T cell receptor repertoires. PLoS Comput Biol 2015;11:e1004503.
